# Techno-economic-environmental evaluation of a solar-hydrogen-battery hybrid system: a real-time case study

**DOI:** 10.1038/s41598-026-54814-4

**Published:** 2026-06-04

**Authors:** Hedra Saleeb, Mohamed F. Baroma, Almoataz Y. Abdelaziz, Rasha Kassem

**Affiliations:** 1 Electrical Department, Faculty of Technology and Education, Sohag University, Sohag, 82524 Egypt; 2 Faculty of Engineering, Ain Shams University, Cairo, 11517 Egypt; 3 Faculty of Engineering, Future University in Egypt, Cairo, 11835 Egypt

**Keywords:** Green hydrogen, Solar PV, Hybrid energy storage, Techno-economic analysis, Climate, Egypt, Energy and society, Energy science and technology, Engineering

## Abstract

The global shift toward net-zero carbon emissions requires flexible, multi-source energy systems capable of overcoming disruptions in renewable energy sources. This study presents a comprehensive technical, economic and environmental assessment of a hybrid energy system designed for the Faculty of Technology and Education at Sohag University, Egypt. The research evaluates three operational scenarios, involving the integration of the utility grid (UG), photovoltaic (PV) cells, a battery energy storage system (BESS), and a green hydrogen production subsystem consisting of an electrolyzer, hydrogen storage (H_2_), and fuel cells (FC). Scenario 1 (PV/BESS/UG) serves as the baseline configuration, achieving a renewable fraction of 74.7% but maintaining significant dependence on the electrical grid. Scenario 2 (PV/FC/H2/UG) demonstrated the economic infeasibility of a hydrogen subsystem configured to operate on a daily charge–discharge cycle rather than functioning as a long-duration or seasonal storage system; the optimization results favored grid electricity over fuel cell dispatch. Scenario 3 (PV/FC/BESS/H_2_/UG) emerges as the most effective configuration. Despite exhibiting a higher net present cost (NPC: 823,477 USD) and a levelized cost of energy (LCOE: 0.0832 USD/kWh), it achieved a renewable fraction of 75.7% and ensured nearly 100% supply reliability with negligible unmet electrical load. The results indicate that the integration of BESS for short-term response and H_2_ for long-term energy reserve provides a strategic energy buffer capable of mitigating the effects of solar PV power outages and grid instability.

## Introduction

The world today faces a chronic problem that requires immediate intervention, as it has significant and tangible repercussions across various fields that differ in description but converge in impact. This problem is global warming resulting from fossil fuels, which has led to a perceptible climate change with a broad influence on life in different regions, regardless of their climatic nature, whether coastal or desert^1,2^. This issue appears more intensely in hot regions as a natural characteristic of their climate, where the total heat becomes substantial due to global warming. Combined with population density and the emission of greenhouse gases from the most commonly used machines in the current era, such as traditional cars that remain widespread worldwide despite ongoing efforts to make them environmentally friendly^3^. By narrowing the focus to specific sites within these regions that possess the aforementioned characteristics, the focus shifts toward facilities and loads of a pivotal nature in society, such as hospitals, universities, and sensitive government agencies. These sites are of particular importance, as a high level of reliability and continuity of energy supply must be ensured, in line with the global trend and consistent with Egypt Vision 2030 for a net-zero emissions public infrastructure that relies on unconventional sources for energy production and storage, and reduces dependence on the electricity grid. This presents a challenge in such sites, especially those with harsh climatic conditions, such as Upper Egypt^4^. From this perspective, this research paper seeks to evaluate the performance of storage systems based on renewable energy and to develop a set of scenarios for supplying an academic building in the climate of Sohag in Upper Egypt. The aim is to examine the effectiveness of storage methods in terms of balancing reliability and economic feasibility, and to evaluate these scenarios to provide a clear insight into their degree of dependability and subsequent impacts. Therefore, let us review the available literature on this subject to identify the specific gaps and the rationale behind this research. This study offers several key contributions, providing clear insights into the system degree of reliability.

- Integrates techno-economic optimization with grid validation of the IEEE 33-bus system, bridging the gap between system sizing and grid-level performance analysis.
- Provides a detailed analytical interpretation of system behavior, including the interaction between short-term (batteries) and long-term (hydrogen) storage mechanisms.
- Incorporates climatic conditions specific to the Upper Egypt hyper-arid environment, which are underrepresented in the literature.
- Critically assesses not only optimal configurations but also their practical and economic viability, with a particular focus on cases where increased reliance on renewable energy sources does not justify higher costs.

Following this introduction, the manuscript is structured as follows: “Literature review” section provides a comprehensive literature review on hybrid microgrids, green hydrogen production technologies, and storage integration, identifying the specific research gaps addressed by this study. “System description and methodology” section describes the system methodology, including the characterization of the selected site in Sohag, Upper Egypt, the detailed load profiling of the academic building, the technical modeling of all system components (PV, battery, electrolyzer, hydrogen storage, and fuel cell), the optimization framework, dispatch strategies, and the economic modeling assumptions. “Results and discussion” section presents the results and discussion, offering a comparative techno-economic analysis of the three proposed grid-connected hybrid energy scenarios. “Validation and modeling” section validates the optimal system configuration through power flow analysis using the IEEE 33-bus radial distribution network, assessing the impact on grid voltage stability. Finally, “Conclusion and future work” section concludes the study by summarizing the key findings, highlighting the trade-offs between economic feasibility and energy resilience, and outlining the implications for sustainable energy infrastructure.

## Literature review

Relying on hybrid microgrids has become an effective strategy in the recent era. Their use has been observed in many locations, particularly to address the current global crisis, reducing carbon emissions alongside other goals such as providing autonomy and reliability while achieving economic feasibility in remote coastal areas, such as Hatia Island in Bangladesh^5^. This study achieved a cost of energy (COE) of 0.0214 USD/kWh, which is an excellent cost compared to^6^, which achieved a COE of 0.34 USD/kWh in a proposal aimed at achieving stability in a rural community within the same geographical sector of Bangladesh, albeit with a different coastal environment. Despite this significant difference in COE, each has its justifications. However, moving from this coastal country to Africa, specifically the Woudi region in Cameroon, reached a COE of 0.256 USD/kWh when using lithium-ion batteries in a hybrid scenario in that semi-desert region. This system relied on a hybrid configuration of sources varying between geothermal, biogas, wind, and photovoltaic (PV) energy^7^. It is worth noting that reviewing a study that combines economic evaluation and climatic diversity for the performance of a hybrid system relying on solar energy and fuel cells to feed loads while integrating with the local grid is of paramount importance. This study addressed this issue, which previous studies had overlooked^8^. This study combined these criteria by examining climatic variations across five different locations in the United States^9^. The study was based on the economic evaluation of a system consisting of bifacial solar cells with a hydrogen system integrated with the grid. The results were intriguing, showing that the use of bifacial solar cells reduced the COE from 0.034 to 0.029 USD/kWh, thanks to the optimal utilization of solar radiation. However, what the study did not mention was the effectiveness of the lifespan of these relatively new panels, how the system would perform if a fast-response storage element like batteries were added instead of total reliance on the relatively slow-response hydrogen system for long-term storage, and it did not specify the rationale behind the load selection, whether it was random or followed a specific philosophy^10^.

Since hydrogen represents the breakthrough of the recent era and the compass of scientific research,^11^ stated that the integration of solar and wind energy as renewable sources results in modest hydrogen production, with a price of 2 USD/kg. Meanwhile,^12^ mentioned that the cost of producing hydrogen using wind energy reaches 8.6 euros/kg and may reach 11.17 euros/kg, considering transportation and storage costs in its liquid state. Conversely,^13^ stated that the average cost of producing hydrogen from renewable energy, solar or wind, ranges between 1 and 5 USD, considering capital and operational expenditures (CAPEX and OPEX), which aligns with global estimates for hydrogen production. The energy consumption rate in electrolyzers for hydrogen production is high compared to concentrated photovoltaic sources. Also, the efficiency of hydrogen production in wind farms is higher than in photovoltaic plants in the Morocco region^14^. Since there are many technologies for hydrogen production using renewable energy, a comparative analysis was conducted between different technologies and concluded that alkaline electrolysis (AEL) is the most mature among available technologies^15^. The justifications included its longevity, as it has been a utilized technology for over a century, providing single-stack capacities up to 6 MW^16^. Notably, AEL was designed for stationary applications and must be adapted to the required application; its response speed is relatively modest^17^. This prompted manufacturers to develop ton exchange membrane electrolysis (PEMEL), as flexibility in applications became a significant demand in recent years, though the capacity challenge was relatively less of an obstacle. PEMEL is superior to AEL in several aspects, including the possibility of pressurized operation, flexibility, and shorter startup times, especially in cold conditions, with the ability to vary the production rate across the full load range^18^. In stark contrast, solid oxide electrolysis (SOEL) remains under testing; despite its unique advantages, it still lacks an accurate estimation of lifespan, making it experimental compared to the prevalence of AEL and PEMEL^19^. The most promising technology with a bright future is PEMEL, because hydrogen production via water electrolysis is an environmentally friendly option where the only byproduct is oxygen, unlike traditional methods. Despite this importance, the total value of global water electrolysis accounted for only 4% of industrial hydrogen in 2019^20,21^.

The thermochemical and gasification decomposition represent two widely applicable and economic options for hydrogen production, while traditional methods maintain dominance at costs ranging from 1.34 to 2.27 USD/kg^22^. Biological methods are on the path of development with a promising future, but still require more research to prove their effectiveness. In contrast, low conversion efficiency and high capital costs remain obstacles for renewable power sources in producing green hydrogen. This is the global trend and the research area where the world seeks to reap benefits, and renewable methods will eventually dominate^23–26^. On the path of developing hydrogen production strategies, they built a strategy to maximize hydrogen production, increasing it by up to 30% through the integration of concentrated photovoltaic thermal (CPVT) with an organic Rankine cycle (ORC) to maximize electricity production from solar radiation and heat^27^. Heat was the Trojan horse the researcher utilized, not sufficing with the solar output of the panels alone, but also benefiting from solar heat. Using reflective mirrors, it reached 160 suns, resulting in an electricity output of 1152 W. The study also reached an electrolyzer efficiency of 57%. While the proposed system is relatively complex, this does not negate the innovation in reducing waste and activating utilization, especially from renewable energy, which is the future of the Earth^28–33^. What previous literature overlooked can be divided into two axes: First, the study of integration between different storage systems, such as batteries and green hydrogen, demonstrating the effectiveness of each method individually and the benefit of using both technologies together in a single grid integrated with PV cells. This aims to clarify the advantages and disadvantages of using these technologies in hot climates, such as Sohag, Egypt, where the probability of battery failure increases^34,35^. The second axis is the study of the effectiveness of these technologies in terms of reliability and economic feasibility on real-time academic and research loads, such as the Faculty of Technology and Education at Sohag University. This load is divided into sections, including emergency loads like research laboratories that cannot tolerate power interruptions due to ongoing tests and experiments^36,37^, and administrative and teaching loads, which are also vital and must be supplied with reliability to ensure the continuity of the educational process^38,39^.

## System description and methodology

### Site characterization

**Fig. 1:**
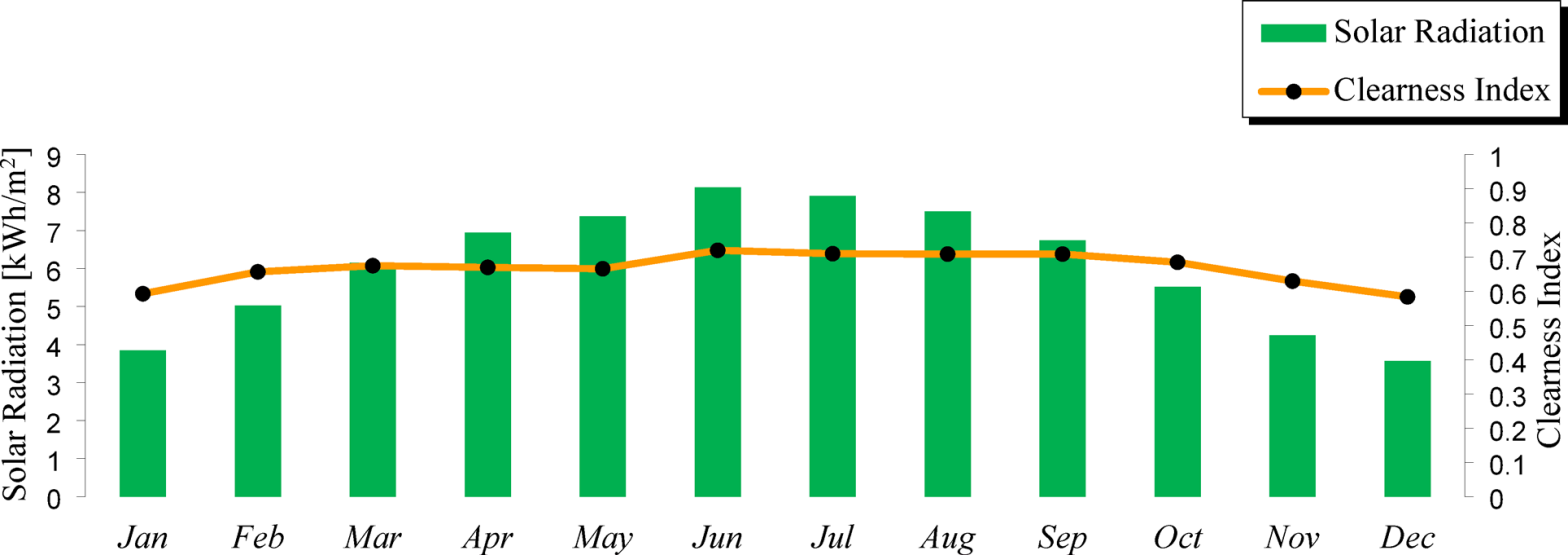
Monthly averages of GHI data in the selected location.

The present study evaluates the educational load profile of the Faculty of Technology and Education at Sohag University. It is imperative to clarify that the investigated facility is geographically decoupled from the main university campus, situated at the coordinates 26.55° N latitude and 31.69° E longitude. Table 1 shows the typical monthly values of global solar radiation over Sohag City^40,41^. For evaluation, the most challenging month is chosen to make sure that the system will function all year long. In Sohag City, in December, the median daily sunlight is 6 h. The solar radiation data for the proposed site were obtained from the NASA surface meteorology and solar energy database website. As illustrated in Fig. 1, this specific regional positioning in Upper Egypt is characterized by a high global horizontal irradiance (GHI), providing a fertile ground for intensive PV penetration. The chart confirms that there are a lot of solar energy events at this location, particularly in the summer, when the mean solar radiation in June was 8.13 kWh/m^2^/day^42,43^.

**Table 1:** Average monthly values of daily solar radiation over Sohag City.

Month	Clearness index	Solar radiation [kWh/m^2^]*	Temperature [°C]
January	0.593	3.850	12.420
February	0.657	5.020	14.090
March	0.675	6.150	18.240
April	0.670	6.940	23.680
May	0.666	7.370	28.100
June	0.720	8.130	30.390
July	0.710	7.910	31.210
August	0.709	7.500	30.810
September	0.709	6.740	28.580
October	0.685	5.520	24.640
November	0.630	4.240	18.840
December	0.584	3.570	13.980

*NB: Monthly averages for global horizontal solar radiation over 22 years (July 1983–June 2005).

NB: Monthly averages for air temperature over 30 years (January 1984–December 2013).

To ensure the reliability of the meteorological inputs, the NASA POWER data were validated through comparison with ground-based measurements and published regional datasets. Specifically, the annual average global horizontal irradiance (GHI) and temperature values were cross-checked against reported values for Upper Egypt in the literature and national meteorological records. The obtained annual average GHI (~ 5.5–6.0 kWh/m^2^/day) and average ambient temperature (~ 25–27 °C) were found to be in close agreement with previously published studies, with deviations within an acceptable range (typically less than 5%).

### Load characterization

#### Administrative load

**Fig. 2:**
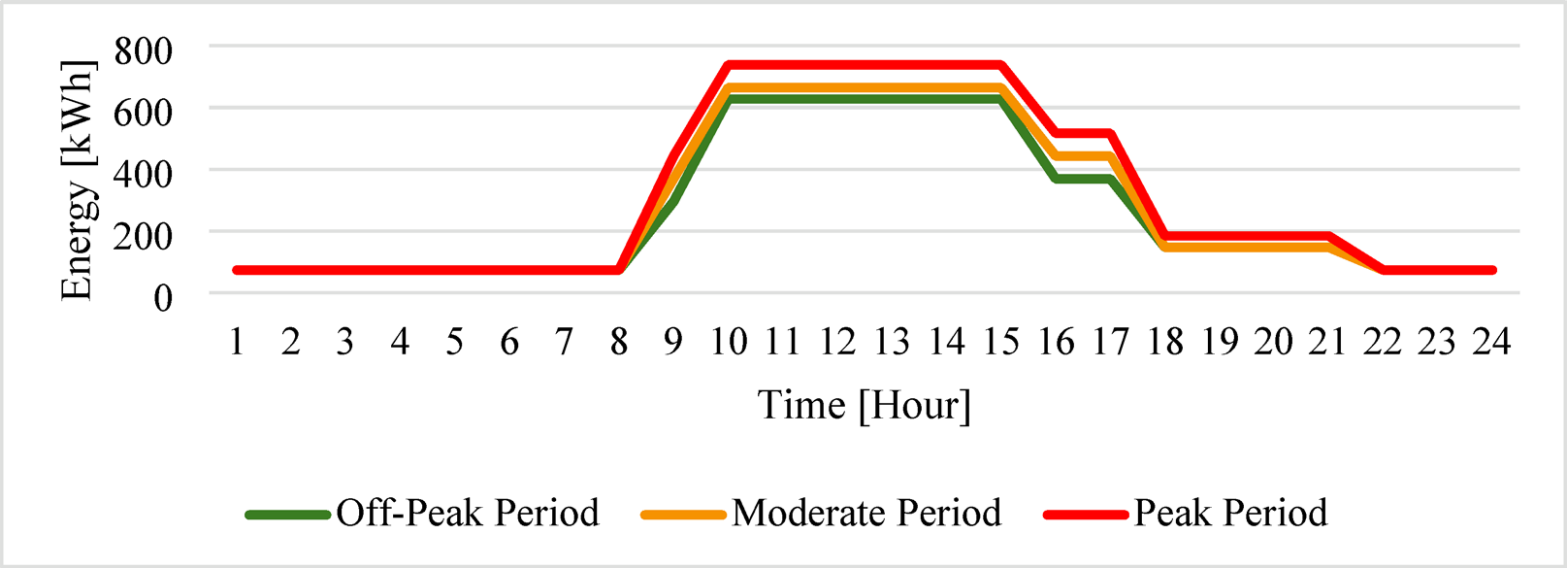
24-h average consumption profiles of the administrative building.

**Fig. 3:**
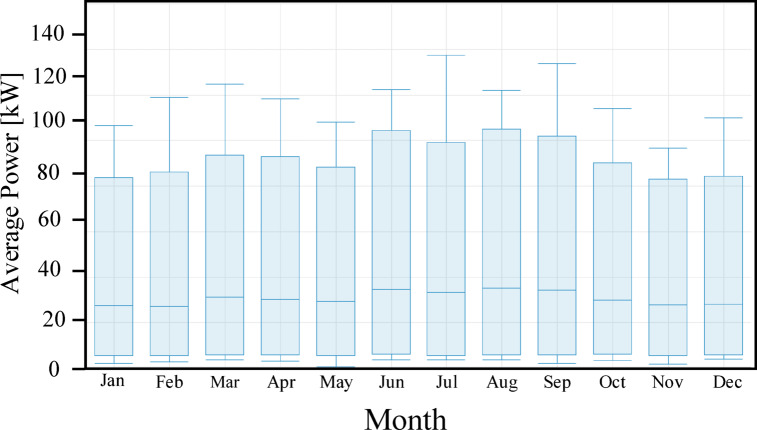
Monthly distribution of the administrative load, showing average demand and seasonal variation.

The dynamic morphology of the administrative load for the Faculty of Technology and Education is illustrated in Fig. 2, providing a diagnosis of the hourly and seasonal consumption trajectories. A stable base load (73.8 kW at night) with a significant daytime variation (627.3–738 kW) due to lighting, air conditioning, and office equipment usage during working hours. Unlike erratic industrial loads, the administrative demand demonstrates a consistent stability between 10:00 and 15:00, indicating a highly disciplined and predictable energy consumption pattern during official working hours. Figure 3 isolates the impact of Sohag climate through the vertical expansion of the bar groups during the summer months (June–September). This stratification provides clear proof of the thermal-electrical coupling, where peak demand stretches vertically in response to rising ambient temperatures.

#### Teaching load

**Fig. 4:**
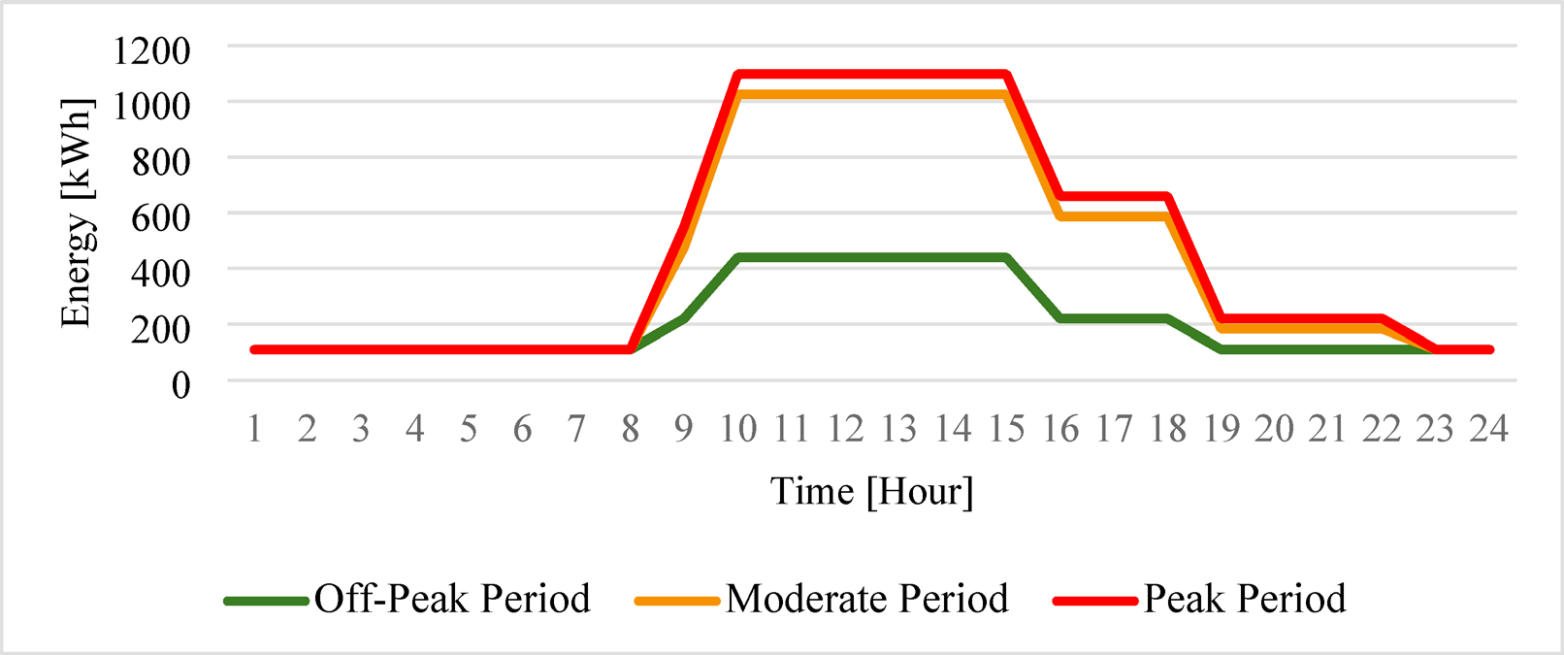
24-h average consumption profiles of the teaching building.

The representation of the hourly electricity consumption for the teaching building, as illustrated in Fig. 4, provides a clear diagnosis of the diurnal load transitions. Figure 4 illustrates a steep morning gradient starting at 08:00, followed by a phased evening ramp-down starting at 15:00. Figure 4 confirms a significant energy pulse during the daylight hours, where the consumption rapidly scales from the 109.8 kW Baseline to a sustained plateau of 1098.00 kW. This tenfold expansion is a visual indicator of the heavy pedagogical activities, particularly the simultaneous operation of laboratory equipment and lecture hall HVAC systems. A pivotal observation from Fig. 4 is the near-perfect synchronization between the building peak demand (10:00 to 15:00) and the peak solar irradiation window in Sohag. This graphical alignment strongly supports the technical feasibility of the PV-HESS integration, as the highest energy requirements can be directly met by solar generation, thereby reducing the stress on the hydrogen fuel cell during daylight hours.

**Fig. 5:**
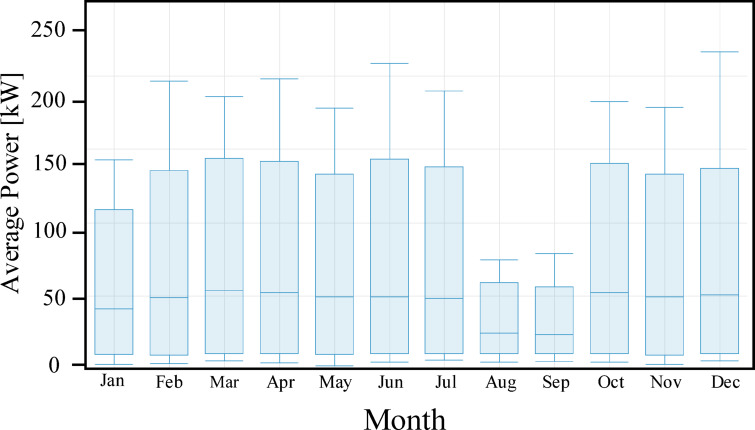
Monthly distribution of the teaching load, showing average demand and seasonal variation.

To evaluate the operational stability and peak demand distribution of the teaching building, a statistical analysis was conducted as illustrated in Fig. 5. This perspective provides a rigorous assessment of the seasonal load transitions, which are a key driver for the HESS sizing. The chart highlights a rigid consistency in peak demand across most months, with the exception of the significant load void observed in August and September. During these months, the peak envelope is visibly suppressed to 439.2 kW, illustrating the found impact of the academic recess on the facility total energy footprint. In months like January, the evening transition is more noticeable, with the load stabilizing at 219.6 kW or 164.7 kW by 18:00, providing a critical visual metric for the dispatch logic of the BESS during the post-sunset period.

#### Load assessment

The annual electrical demand of the Faculty of Technology and Education was estimated based on the hourly load profile of the administrative offices, classrooms, laboratories, and auxiliary services. The total annual electricity consumption reached approximately 6048 MWh/year, with an average daily demand of 16,570 kWh/day. The load profile exhibited significant daytime variation due to teaching activities, laboratory operation, lighting, air conditioning, and office equipment usage during working hours. The peak demand occurred during summer months and was estimated at approximately 1836 kW, while the minimum nighttime load was considerably lower because only security lighting and essential services remained operational. This load magnitude explains why HOMER selected PV capacities in the range of 306–308 kW across the investigated scenarios. The selected PV size is approximately 1.4–1.6 times the peak load, which is consistent with the need to satisfy daytime demand while also generating surplus electricity for battery charging and hydrogen production. Similarly, the battery autonomy and hydrogen storage capacities were sized to ensure supply continuity during evening hours, cloudy conditions, and seasonal fluctuations in solar generation.

### Technical modeling of components

#### Solar photovoltaic array

The PV system performance was modeled in HOMER by incorporating standard module parameters, including efficiency, temperature coefficient, and nominal operating cell temperature (NOCT). The PV module efficiency was assumed to be 18%, representing commercially available mono-crystalline silicon panels. The temperature coefficient of power was taken as − 0.4%/°C, indicating the reduction in output power with increasing cell temperature. The nominal operating cell temperature (NOCT) was assumed to be 45 °C, which reflects typical operating conditions under 800 W/m^2^ irradiance, 20 °C ambient temperature, and 1 m/s wind speed. These parameters were incorporated into the temperature-dependent PV output model in HOMER, where the cell temperature is estimated as a function of ambient temperature and solar irradiance. The negative temperature coefficient is particularly important under Sohag high ambient temperatures, as it leads to a reduction in PV efficiency during peak summer conditions. In addition, a power derating factor was applied to account for losses resulting from dust accumulation, wiring mismatch, and inverter inefficiency. The instantaneous power output (${P}_{PV}$) is governed by the following expression:

1 $${P}_{PV}={Y}_{PV}{F}_{PV}\left(\frac{{G}_{T}}{{G}_{T,STC}}\right)[1+{\alpha}_{P}({T}_{C}-{T}_{C,STC})]$$

where:

- ${Y}_{PV}$: Rated capacity of the PV array under standard test conditions (STC) [kW].
- ${F}_{PV}$: PV derating factor [dimensionless].
- ${G}_{T}$: Solar radiation incident on the PV array in the current time step [$\mathrm{K}\mathrm{W}/{\mathrm{m}}^{2}$].
- ${G}_{T,STC}$: Incident radiation at STC [$\mathrm{K}\mathrm{W}/{\mathrm{m}}^{2}$].
- ${\alpha}_{P}$: Temperature coefficient of power $[\mathrm{\%}/{\mathrm{C}}^{^\circ }]$.
- ${T}_{C}$: Actual PV cell temperature in the current time step [${\mathrm{C}}^{^\circ }$].
- ${T}_{C,STC}$: PV cell temperature under STC [25 °C].

#### Battery energy storage system

In this study, the lithium-ion battery was assumed to have a nominal lifetime of 10–15 years or approximately 3000–5000 cycles at a specified depth of discharge. HOMER estimates battery wear based on cumulative charge/discharge energy and schedules replacement when the total throughput limit is reached. The capital cost was assumed to be in the range of 400–600 USD/kWh, while the replacement cost was taken as slightly lower (e.g., 80–90% of the initial capital cost) to reflect future cost reductions. Operation and maintenance (O&M) costs were assumed to be minimal, consistent with lithium-ion technology. Battery operation was further constrained by a minimum state of charge (SOC) of 20% and a maximum SOC of 100% to avoid deep discharge and extend battery lifetime. Round-trip efficiency was assumed to be approximately 90–95%, consistent with typical lithium-ion performance. Lithium-ion technology was prioritized due to its superior energy density and resilience under deep discharge cycles. The dynamic behavior of the SOC is modeled as follows:

2 $$\mathrm{S}\mathrm{O}\mathrm{C}\left(\mathrm{t}\right)=\mathrm{S}\mathrm{O}\mathrm{C}\left(\mathrm{t}-1\right)*\left(1-\sigma \right)+(\frac{{P}_{batt}\left(t\right)*{\eta}_{batt}}{{E}_{nom}})$$

where:

- $\mathrm{S}\mathrm{O}\mathrm{C}\left(\mathrm{t}\right)$: State of charge at the current time step.
- $\mathrm{S}\mathrm{O}\mathrm{C}\left(\mathrm{t}-1\right)$: State of charge at the previous time step.
- $\sigma$: Self-discharge rate of the battery bank.
- ${P}_{batt}\left(t\right)$: Power charged into or discharged from the battery [kW].
- ${\eta}_{batt}$: Round-trip efficiency of the battery system.
- ${E}_{nom}$: Nominal capacity of the battery bank [kWh].

#### PEM electrolyzer and hydrogen storage

A PEM electrolyzer was integrated to convert surplus PV power into green hydrogen. The hydrogen production model presented in Eq. (3) assumes a simplified relationship between the electrical input power and hydrogen output. The electrolyzer efficiency was assumed to be in the range of 65–75% (based on the lower heating value of hydrogen), which is consistent with commercially available alkaline and PEM electrolyzers. The electrolyzer was modeled in HOMER using its built-in performance characteristics, where hydrogen production is calculated as a function of input electrical power and efficiency. The operating pressure of the electrolyzer was assumed to be in the range of 20–30 bar, which is typical for integrated hydrogen production and storage systems. This moderate pressure level reduces the need for additional compression while maintaining safe and practical storage conditions. The mass production rate (${M}_{H2}$) is quantified by:

3 $${M}_{H2}=\frac{{\eta}_{elec}* {P}_{elec}}{HH{V}_{H2}}$$

where:

- ${M}_{H2}$: Mass flow rate of hydrogen produced [kg/hr].
- ${\eta}_{elec}$: Energy efficiency of the PEM electrolyzer.
- ${P}_{elec}$: Electrical power consumed by the electrolyzer [kW].
- $HH{V}_{H2}$: Higher heating value of hydrogen [$\sim$ 39.4 kWh/kg].

#### Fuel cell unit

The FC functions as a zero-emission backup system during nighttime or low-irradiance periods. The output power (${P}_{FC}$) is determined by:

4 $${P}_{FC}={M}_{H2,cons}*LH{V}_{H2}* {\eta}_{FC}$$

where:

- ${P}_{FC}$: Electrical power output of the fuel cell [kW].
- ${M}_{H2,cons}$: Mass of hydrogen consumed by the fuel cell [kg/hr].
- $LH{V}_{H2}$: Lower heating value of hydrogen [$\sim$ 33.3 kWh/kg].
- ${\eta}_{FC}$: Overall electrical efficiency of the fuel cell system.

### Optimization framework and investigated scenarios

The system configuration and component sizing were optimized in HOMER using a techno-economic objective function that minimizes the net present cost (NPC) while satisfying load demand and system constraints. The optimization process considered a set of decision variables including PV capacity (0–350 kW), battery storage capacity (0–500 kWh), electrolyzer capacity (0–100 kW), fuel cell capacity (0–75 kW), and hydrogen storage tank size (0–100 kg). These ranges were selected based on the expected load demand and practical deployment limits for institutional-scale systems. Several technical and operational constraints were imposed during the optimization process. The system was required to meet the electrical load with a maximum allowable unmet load fraction of less than 1%, ensuring high reliability. The battery state of charge (SOC) was constrained between 20 and 100% to prevent deep discharge and extend battery lifetime. For the hydrogen subsystem, the electrolyzer and fuel cell were constrained by their rated capacities and operational efficiencies, while the hydrogen tank storage limits were enforced to avoid overfilling or depletion beyond minimum thresholds. Economic assumptions included a project lifetime of 25 years, an annual discount rate of 8%, and component-specific capital, replacement, and O&M costs based on current market data. Grid electricity tariffs were modeled using a fixed purchase price, while excess electricity sales were either restricted or assigned a lower feed-in tariff, depending on the scenario. A power derating factor was also applied to the PV system to account for real-world losses. Additionally, sensitivity variables such as solar irradiance and load variation were considered to ensure the robustness of the optimization results under changing environmental and demand conditions. The final system configurations reported for each scenario correspond to the optimal solutions identified by HOMER that satisfy all constraints while minimizing lifecycle cost.

### The dispatch strategies implemented in HOMER

The two dispatch strategies were applied:

#### Load following (LF) strategy

In this strategy, the generation system, including PV panels and fuel cells, is operated when needed to meet the instantaneous load demand only. Any excess renewable energy is directed toward charging the battery or producing hydrogen via the electrolyzer. The fuel cell is dispatched only when the combined PV and battery output is insufficient to meet the load, thereby minimizing unnecessary fuel consumption and prioritizing renewable utilization.

#### Cycle charging (CC) strategy

In this strategy, when the generator or fuel cell is activated, it operates at or near its rated capacity. The generated power first meets the load demand, and any surplus is used to charge the battery or support hydrogen production. This approach reduces the frequency of start-stop cycles and can improve component lifetime, but may lead to higher short-term energy production than required by the load. Table 2 illustrates the control logic governing power flow and energy management among system components, prioritizing renewable utilization, energy storage, and grid support to ensure reliable load supply.

**Table 2:** Power flow control logic.

Mode 1	PV → Load	Priority to serve the load	
Mode 2	PV → Battery	Exploiting the surplus	Short-term storage
Mode 3	PV → Electrolyzer		Long-term storage
Mode 4	Fuel Cell → Load	Secondary source when renewable generation and stored energy are insufficient	
Mode 5	Grid → Load	Backup to ensure reliability in all scenarios	

### Economic modeling framework and tariff structure

The economic analysis of the proposed HESS is grounded in the commercial tariff structures set by EgyptERA for the 2024/2025 fiscal year. To ensure the global relevance of the findings and to align with international energy market standards, all financial metrics, including NPC and Levelized Cost of Energy (LCOE), have been converted and analyzed in United States Dollars (USD).

#### Rationale for currency selection

The adoption of the USD as the primary currency for this study is justified by its status as the universal benchmark for international energy transactions and technology development. Since the core components of the system (PV modules, electrolyzers, and advanced battery systems) are predominantly traded in global markets, using a stable international currency mitigates the complexities of local currency fluctuations and provides a standardized basis for comparing the project feasibility with global benchmarks.

#### Tariff conversion and current market rates

As of January 20, 2026, the prevailing exchange rate is approximately 47.50 EGP/USD. Consequently, the EgyptERA commercial tariffs, which range from 1.80 to 2.30 EGP/kWh, have been modeled at an equivalent rate of approximately 0.038–0.048 USD/kWh. This conversion provides a realistic baseline for the operational expenditure (OPEX) and revenue modeling within the HOMER environment.

#### The financial parameters

The financial parameters used in the economic modeling within HOMER were applied as follows:

The project lifetime was 25 years, the real discount rate was 8%, the inflation rate was 2%, and the salvage value was calculated automatically by HOMER based on the remaining useful life of each component at the end of the project term. The real discount rate was used to ensure alignment between cost inputs and economic valuation, in accordance with standard technical and economic analysis practices. HOMER internally considers replacement costs, O&M costs, and salvage values when calculating the NPC and the adjusted energy cost LCOE.

#### Sensitivity analysis

Given the volatility of exchange rates and potential shifts in national energy policies, a Sensitivity Analysis was performed on the electricity tariff variable. This analysis explores the impact of a ± 20% variation in the USD-denominated tariff on the system total NPC. This step is crucial for identifying the switching point where grid-connected solar-hydrogen integration becomes more cost-effective than pure grid reliance, ensuring the robustness of the system design against future economic shifts. Table 3 presents the key variables considered in the sensitivity analysis along with their base values, variation ranges, and underlying rationale. The selected ranges reflect realistic uncertainties in economic conditions, component costs, system performance, and environmental inputs, enabling a comprehensive assessment of the robustness of the proposed hybrid energy system.

**Table 3:** Sensitivity variables and assumed ranges used in the analysis.

Category	Parameter	Base value	Sensitivity range	Variation (%)	Rationale
Economic	Electricity tariff (USD/kWh)	Nominal tariff	× 0.8– × 1.2	± 20%	Reflects uncertainty in grid pricing and future tariff escalation
	Discount rate (%)	8%	6%–10%	± 25%	Captures financial uncertainty and investment risk
	Inflation rate (%)	2%	1%–4%	–	Accounts for macroeconomic variability
PV system	Capital cost (USD/kW)	Base cost	× 0.8– × 1.2	± 20%	Represents market fluctuations and cost reduction trends
	Derating factor (%)	90%	85%–95%	–	Accounts for environmental and system losses
Battery system	Replacement cost (USD/kWh)	Base cost	× 0.8– × 1.2	± 20%	Reflects degradation and future price uncertainty
	Round-trip efficiency (%)	90%	85%–95%	–	Captures performance variability
Hydrogen system	Electrolyzer efficiency (%)	70%	65%–75%	–	Represents the performance range of commercial systems
	Fuel cell efficiency (%)	50%	45%–55%	–	Accounts for part-load and operational variations
	Hydrogen storage cost (USD/kg)	Base cost	× 0.8– × 1.2	± 20%	Reflects uncertainty in storage technologies
Load and resource	Load demand (kWh/year)	Base load	× 0.9– × 1.1	± 10%	Accounts for demand growth or reduction
	Solar irradiance (kWh/m^2^/day)	NASA data	± 10% variation	± 10%	Reflects climatic variability and data uncertainty

## Results and discussion

### Detailed scenarios analysis

**Fig. 6:**
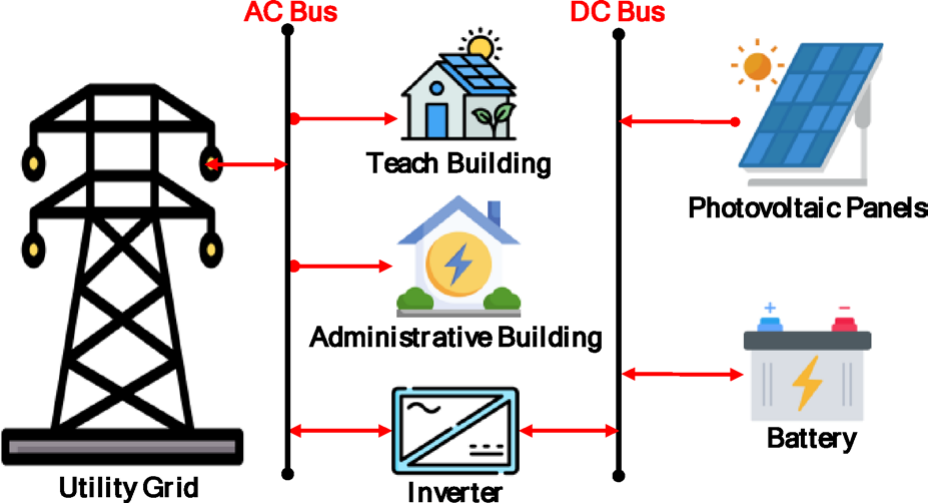
Schematic diagram of Scenario 1.

**Fig. 7:**
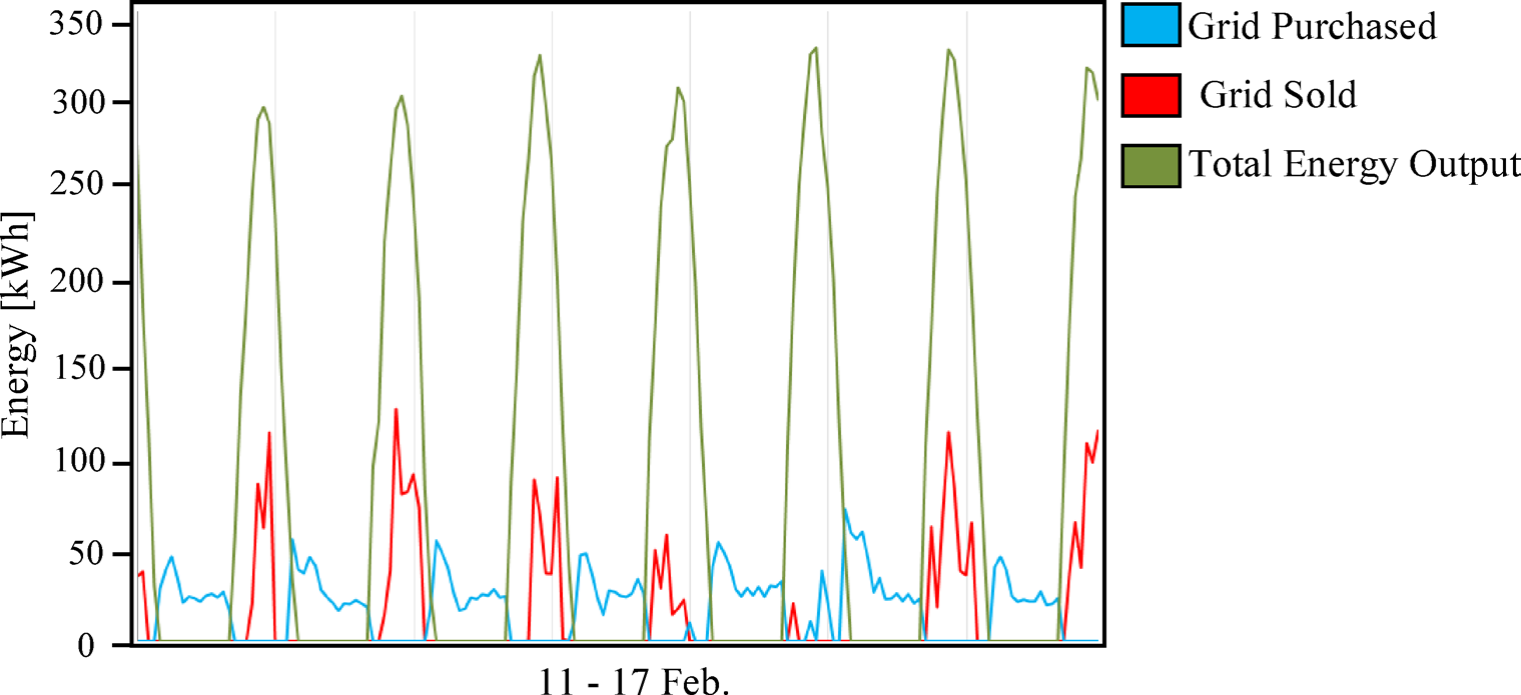
Daily grid energy interaction (purchased vs. sold) for Scenario 1 (PV/BESS/UG) during the period from February 11 to 17.

The comparative techno-economic analysis of the three grid-connected hybrid energy scenarios reveals a clear trade-off between cost-effectiveness and energy resilience. The baseline configuration (Scenario 1: PV/BESS/UG), as shown in Fig. 6, achieved the lowest levelized cost of energy (LCOE) at 0.064 USD/kWh and a net present cost (NPC) of 635,363 USD, establishing it as the economic benchmark. However, its technical performance was constrained by the limited battery autonomy of only 1.05 h. Figure 7 illustrates the relationship between the renewable energy produced by the solar panels used and the amount of energy purchased and sold to the grid in the first scenario in February. As illustrated in Fig. 7, this limitation forced consistent grid purchased during morning 06:00–09:00 and evening 18:00–22:00 peak operational hours, while solar noon surpluses 11:00–14:00 were exported rather than stored. This confirms that battery-only storage is insufficient for achieving energy independence in the Sohag climate.

**Fig. 8:**
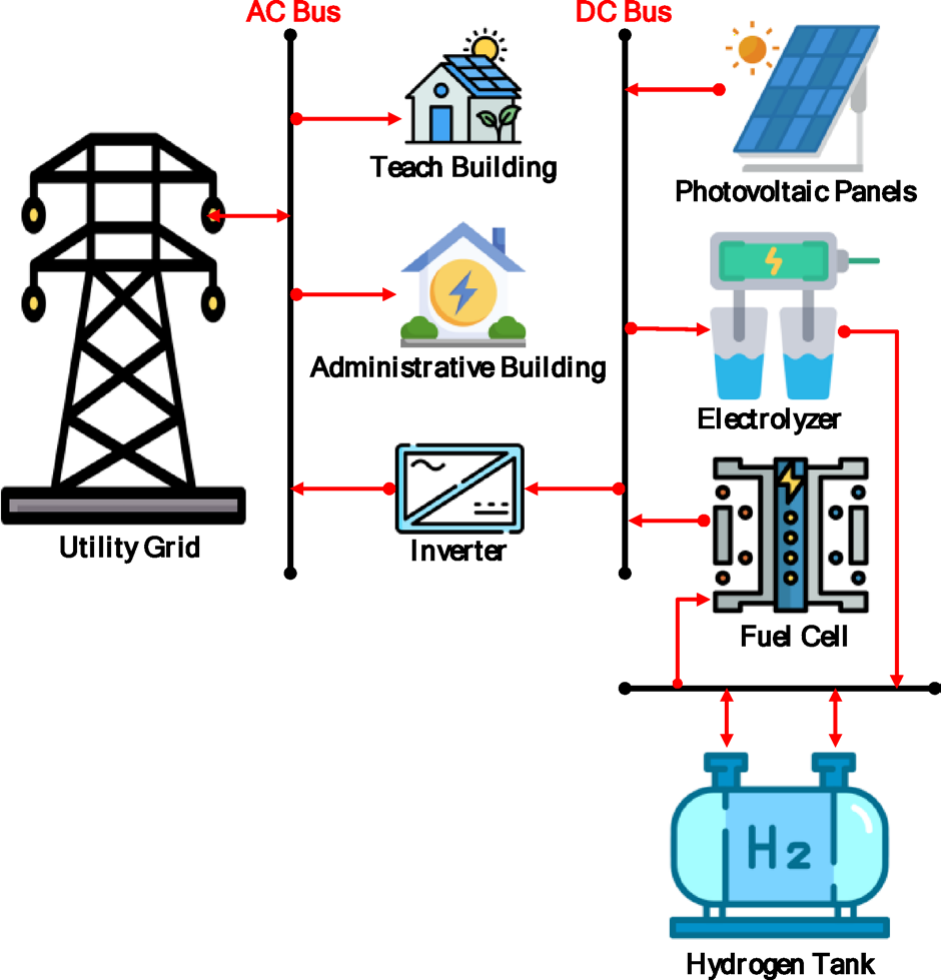
Schematic diagram of Scenario 2.

**Fig. 9:**
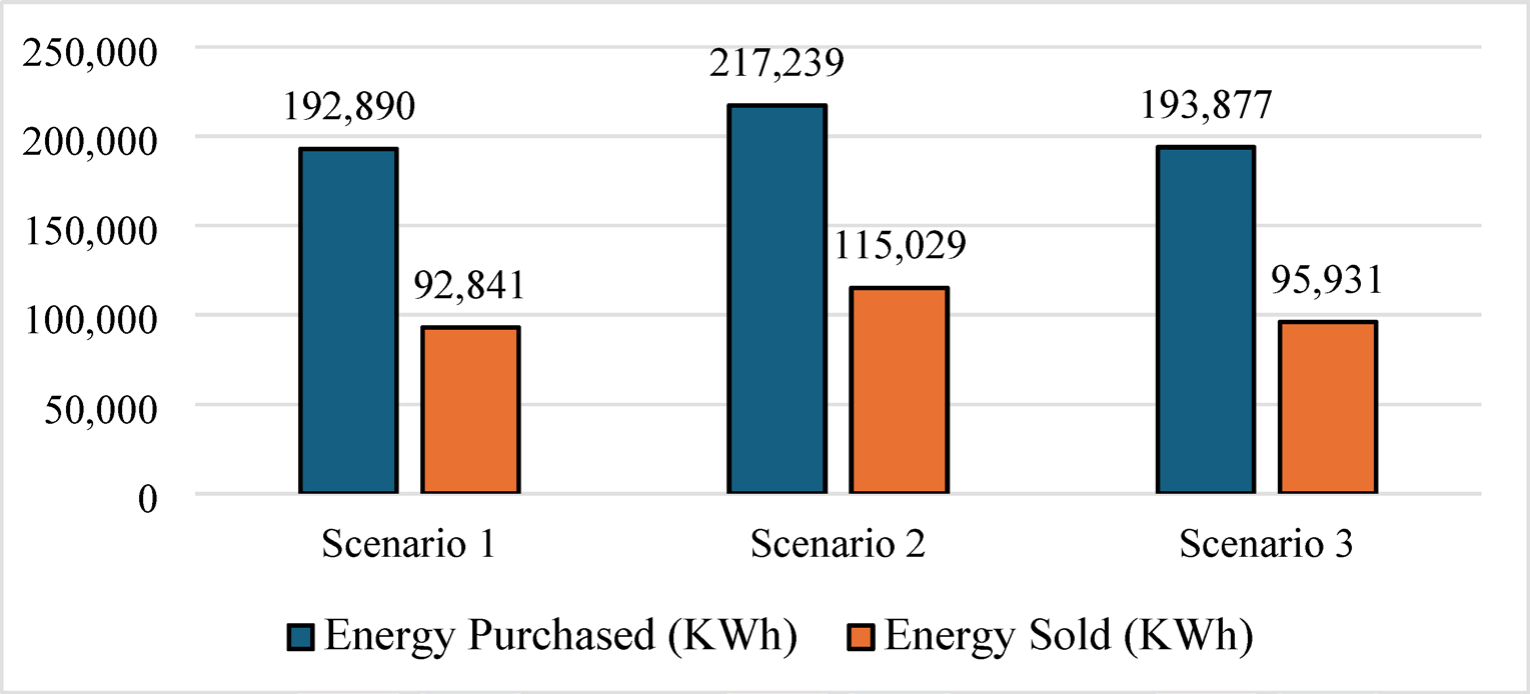
Grid energy dynamics (purchased vs. sold energy).

**Fig. 10:**
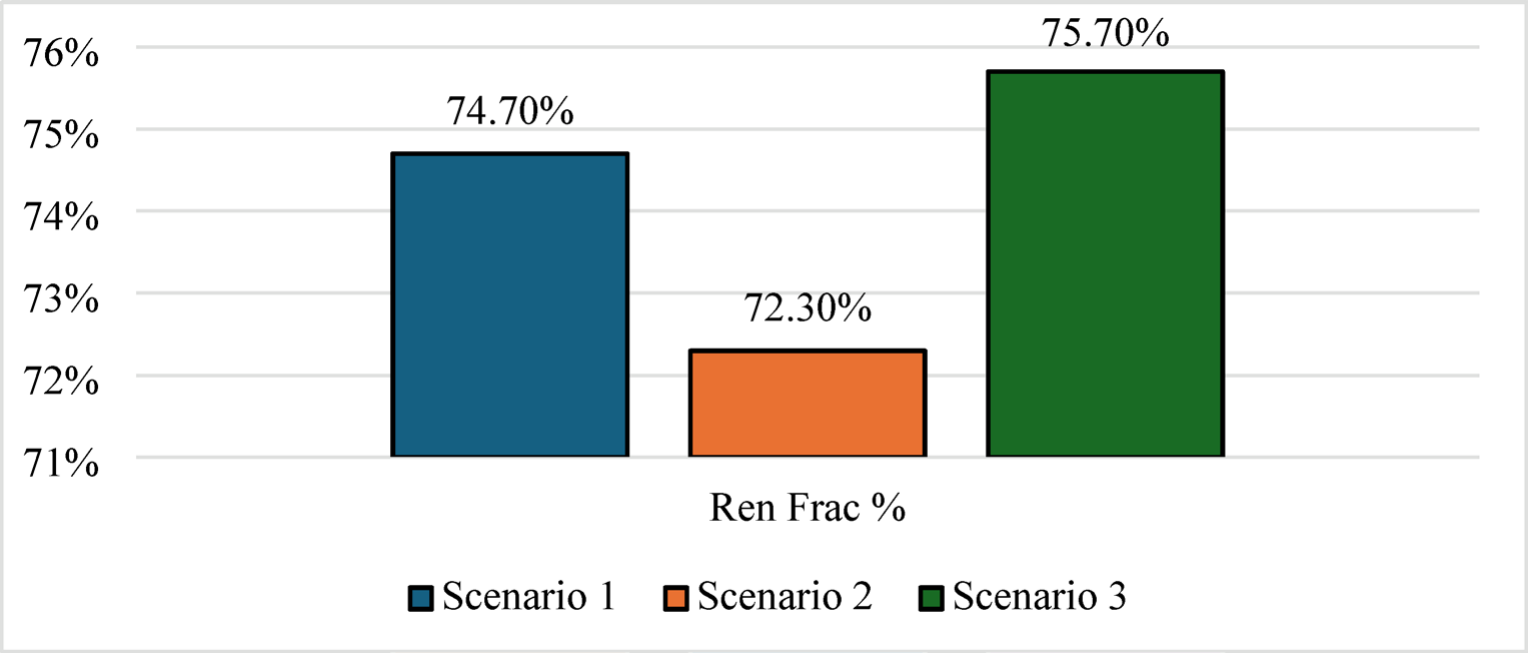
Renewable fraction for the three Scenarios.

**Fig. 11:**
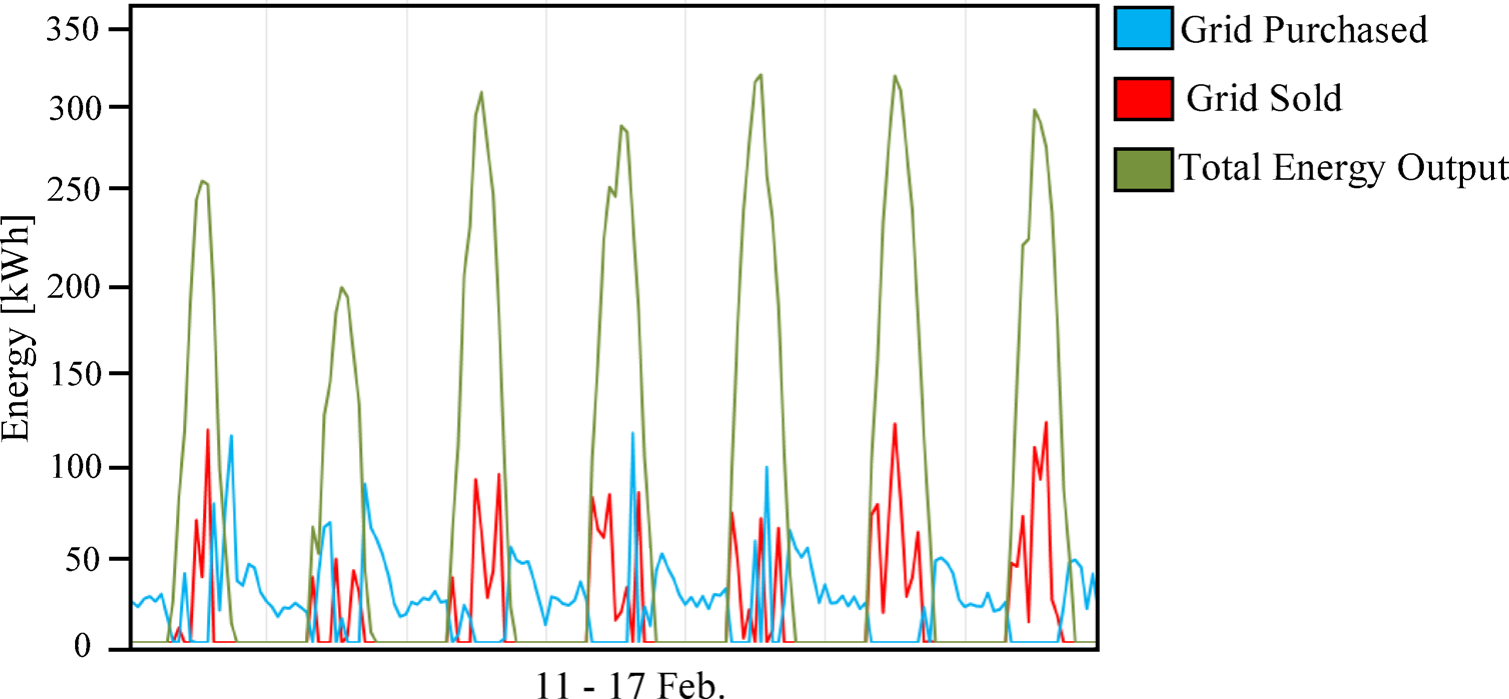
Daily energy balance for Scenario 2 (PV/FC/H_2_/UG) during the period from February 11 to 17.

In contrast, (Scenario 2: PV/FC/H_2_/UG), as shown in Fig. 8, it aims to replace batteries with green hydrogen as a long-term energy carrier. Surprisingly, the HOMER optimizer avoided fuel cell dispatch entirely, favoring grid purchased due to lower short-term costs. As a result, the grid purchased increased by 12.6%, and the renewable fraction dropped to 72.3%, as shown in Figs. 9 and 10. In the absence of batteries, the system cannot leverage stored energy to bridge minor production dips, resulting in a total annual purchased of 217,239 kWh. Figure 11 indicates a substantial increase in energy sold to the grid, reaching an annual total of 115,029 kWh, based on a 100 kW electrolyzer capacity. While a larger electrolyzer could theoretically capture more surpluses, the HOMER optimization identified this specific capacity as the optimal balance point. Increasing the electrolyzer size would disproportionately inflate the NPC beyond the 822,262 USD thresholds with an LCOE of 0.081 USD/kWh. As shown in Fig. 11, although some spilled energy is sold to the grid during peak solar hours, this loss is economically preferable to the higher capital expenditures (CAPEX) required for oversized hydrogen components in this specific scenario.

**Fig. 12:**
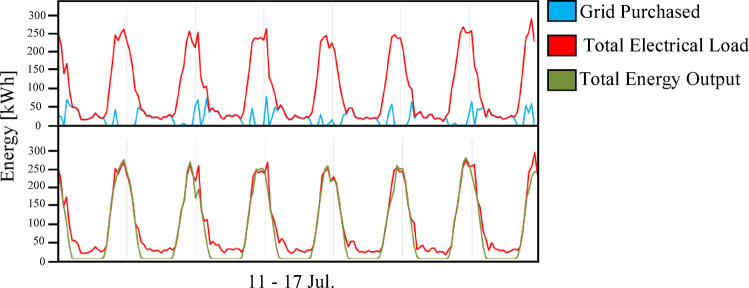
Daily energy distribution (load covered by PV energy vs. energy purchased from the grid) for Scenario 2 during the period from July 11 to 17.

**Fig. 13:**
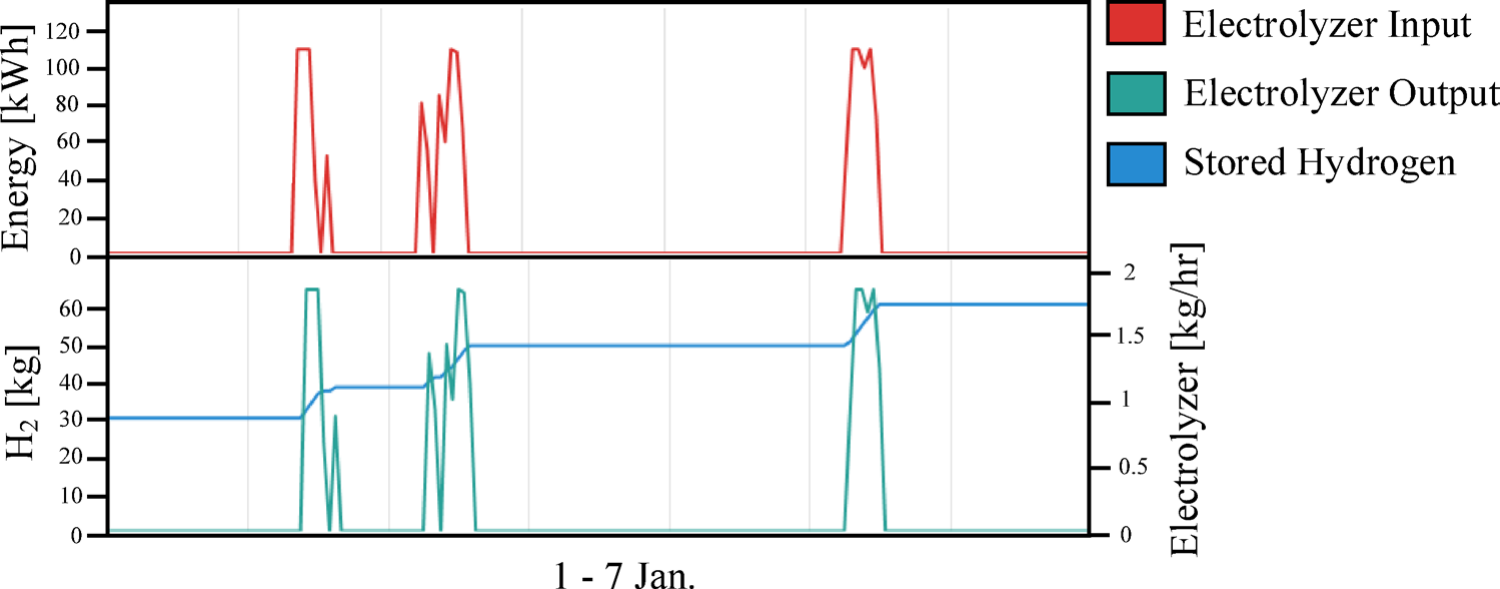
Daily dynamic behavior of the hydrogen subsystem during a representative operational period from January 1 to 7.

Figure 12 illustrates the ratio between the amount of energy used from solar panels to cover the load and the amount of energy purchased from the grid to cover the remaining load. As Fig. 12 shows, the amount of load that the system as a whole has successfully covered, which indicates greater dependence on the grid compared to the previous scenario. Figure 13 illustrates the dynamic behavior of the hydrogen subsystem during a representative operational period. As Fig. 13 shows, the hydrogen subsystem functioned as designed, activating only during solar surpluses. The unit reaches its peak operating capacity of 100 kW, a threshold determined by the HOMER optimization to balance the high capital cost of hydrogen components with the annual energy deficit. During peak intervals, the production rate reaches approximately 1.8 kg/hr, which represents the maximum hydrogen yield for the current system architecture. Although the storage capacity appears to reach its upper limits quickly in some phases, maintaining this 100 kW/tank configuration was found to be the most viable economic solution. According to the optimization results, any further increase in storage volume or electrolyzer size would have inflated the NPC beyond the baseline of 822,262 USD, without a proportional improvement in the Renewable Fraction (72.3%).

**Fig. 14:**
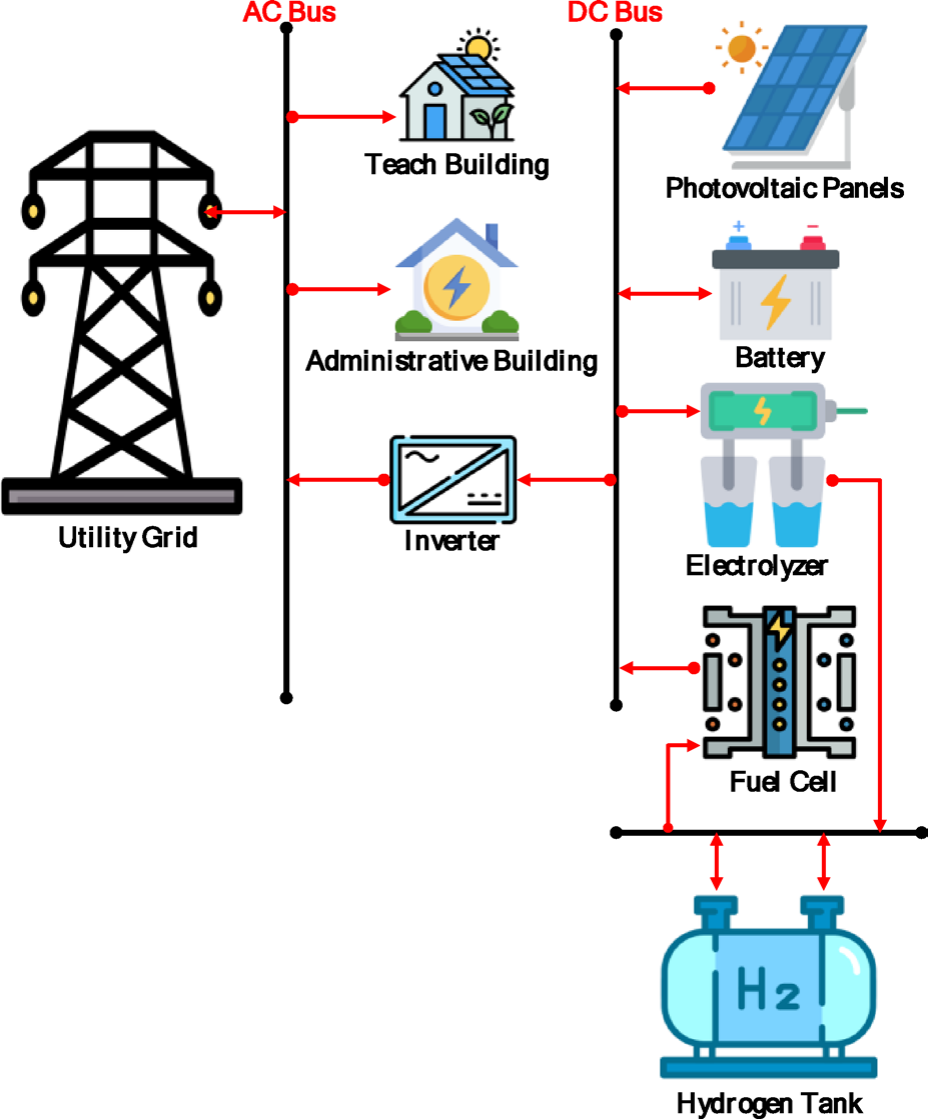
Schematic diagram of Scenario 3.

**Fig. 15:**
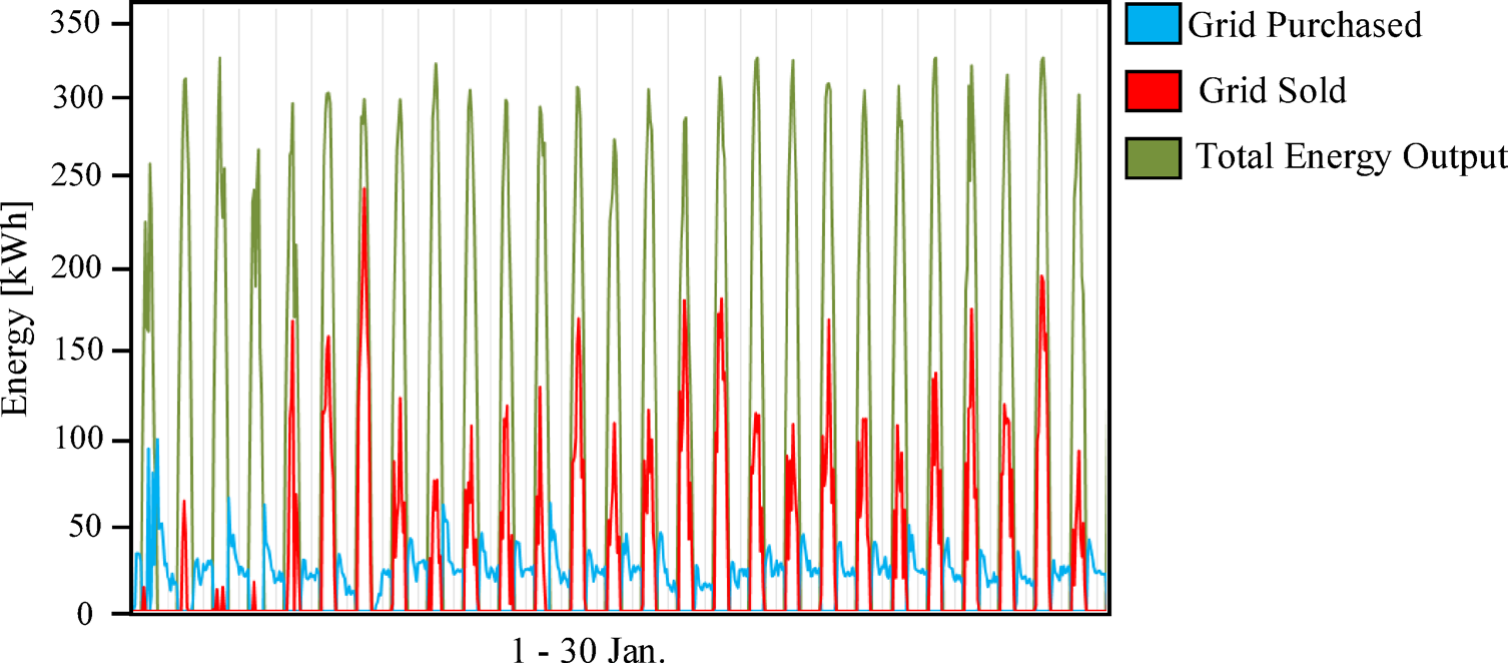
Grid interaction profile for Scenario 3 (PV/FC/BESS/H_2_/UG) during the period from January 1 to 30.

**Fig. 16:**
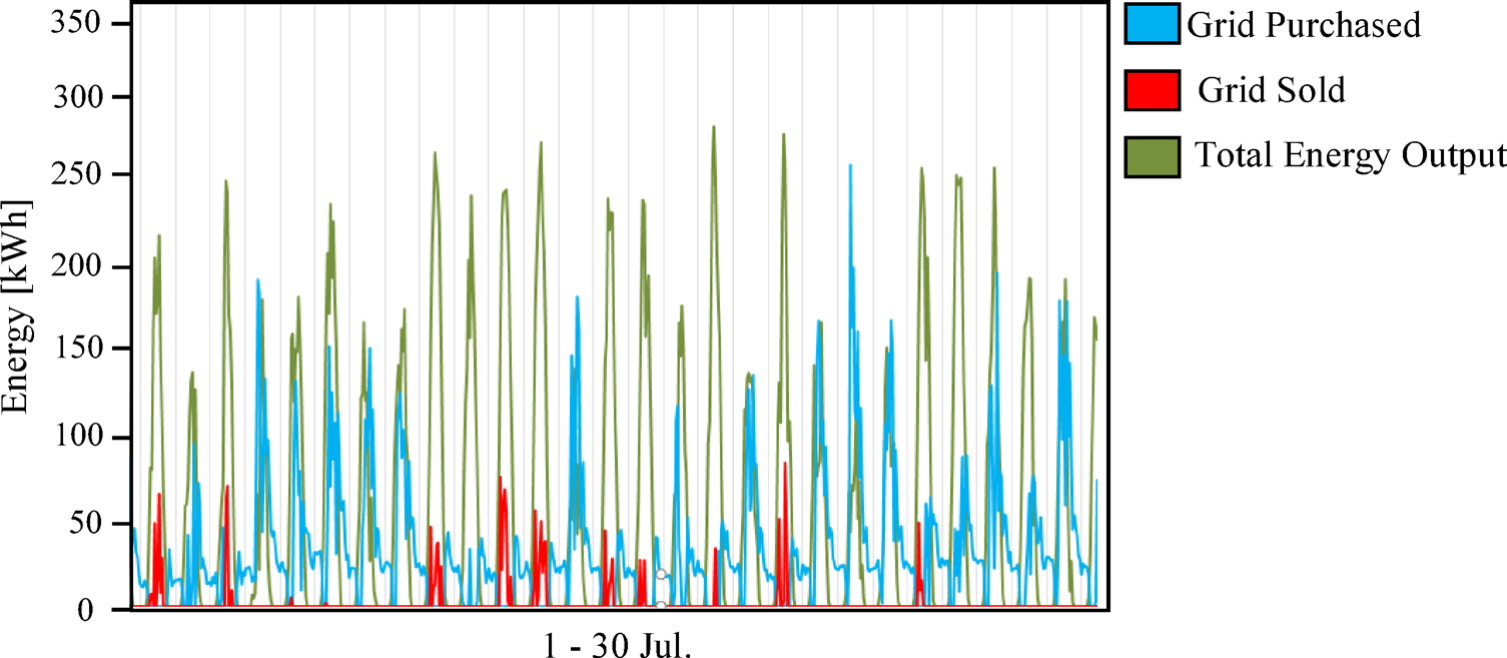
Grid interaction profile for Scenario 3 (PV/FC/BESS/H_2_/UG) during the period from July 1 to 30.

The integrated hybrid system (Scenario 3: PV/FC/BESS/H_2_/UG), as shown in Fig. 14, overcame the limitations of the previous two configurations. By integrating a 100 kWh battery bank for short-term energy storage with a 100 kW electrolyzer and 75 kW fuel cells for long-term energy storage, the system achieved the highest percentage of renewable energy (75.7%) and near-perfect reliability. Figures 15 and 16 illustrate the system interaction with the utility grid during typical winter and summer months. Figures 15 and 16 demonstrate seasonal stability: in January, solar production was optimal with low grid dependency, while July showed reduced PV output due to high-temperature effects on panel efficiency, yet the hybrid buffer compensated effectively. Figure 16 further confirms that energy sold to the grid is lower than in prior scenarios because surplus solar energy is preferentially converted to hydrogen.

**Fig. 17:**
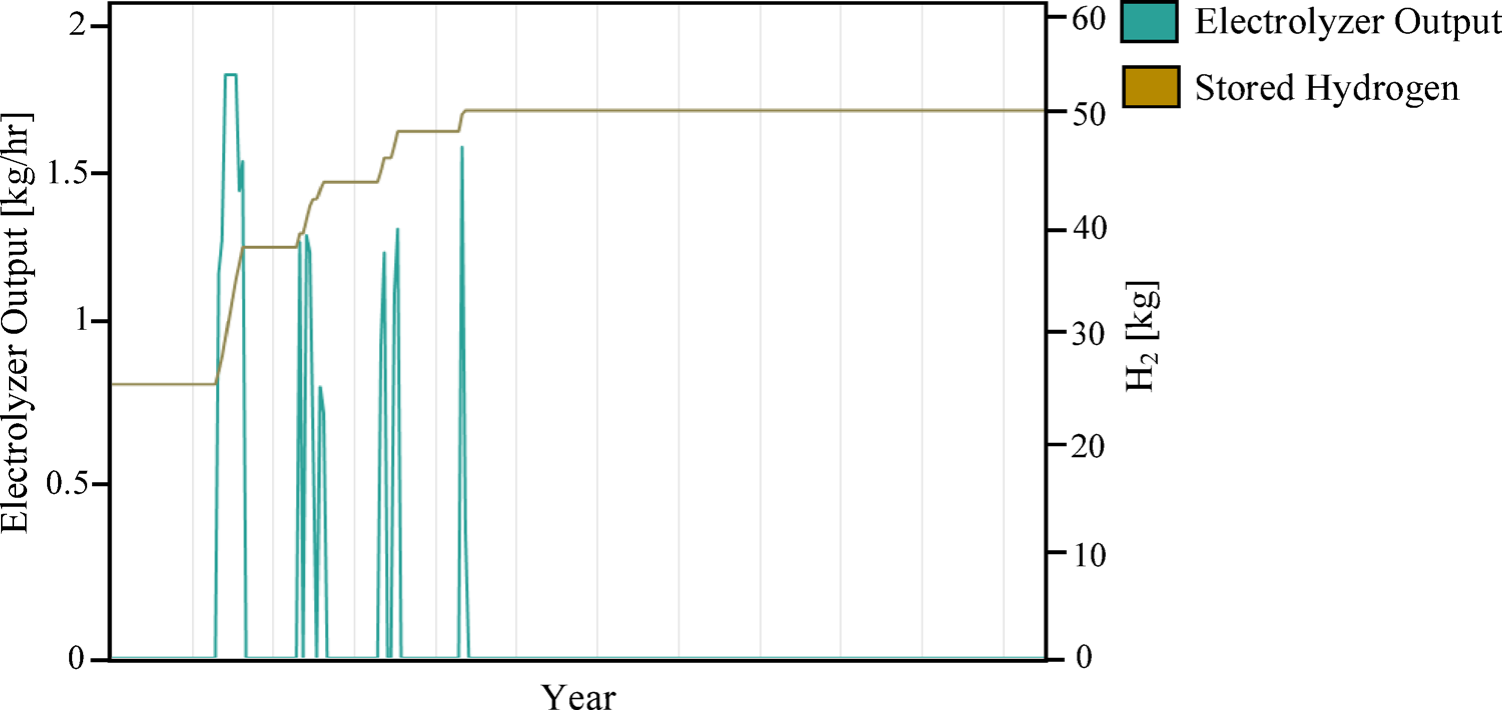
Annual hydrogen subsystem dynamics for Scenario 3 over the year.

**Fig. 18:**
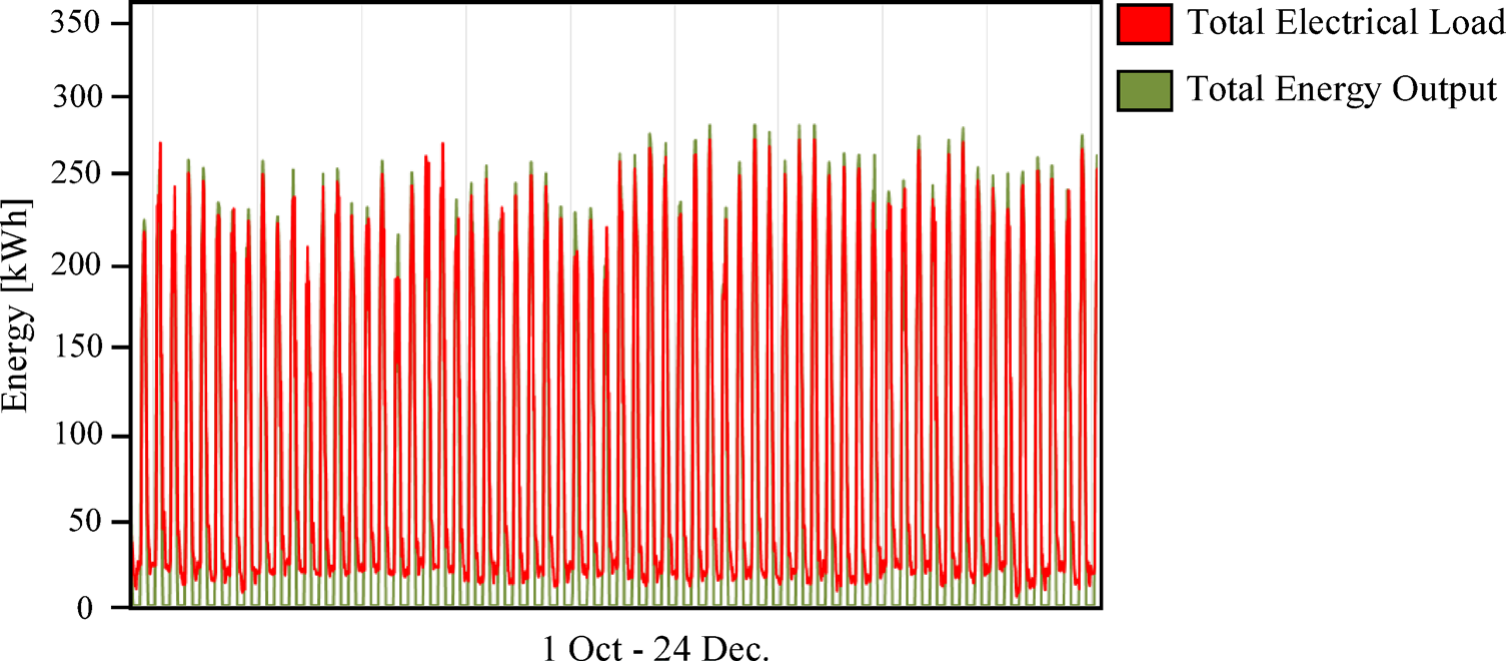
Annual total electrical load profile for Scenario 3 (PV/FC/BESS/H_2_/UG) during the period from October 1 to December 24.

Figure 17 quantifies this success, showing a leap in annual hydrogen production to 1354 kg/year (versus 422 kg/year in Scenario 2), with production peaks reaching nearly 4.5 kg/hr. The synergy between battery and hydrogen subsystems is evident in the smooth, continuous production pulses. More importantly, Fig. 18 confirms the system robustness by calculating the Loss of Power Supply Probability (LPSP) and Loss of Load Probability (LOLP) for Scenario 3 on an hourly basis over the full year. LPSP is defined as the ratio of total unmet electrical load to total load demand, while LOLP represents the fraction of hours in which a deficit occurs regardless of magnitude. For the integrated hybrid configuration (PV/FC/BESS/H_2_/UG), LPSP was effectively zero (i.e., below the simulation tolerance), and the LOLP was 0.00%, indicating that no hour of the year experienced any load interruption. These indices confirm that the system achieves a practical 100% supply reliability under the modeled conditions.

**Fig. 19:**
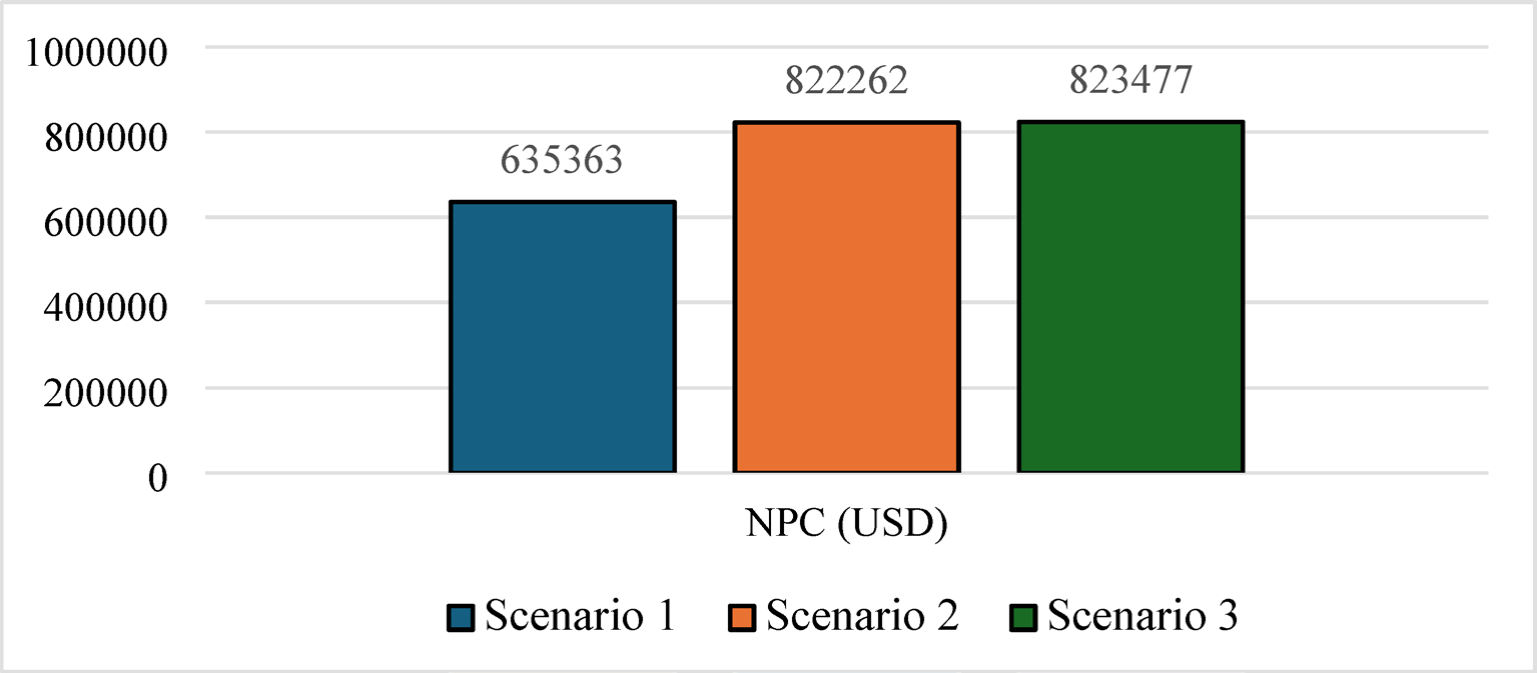
NPC comparison for the three scenarios.

**Fig. 20:**
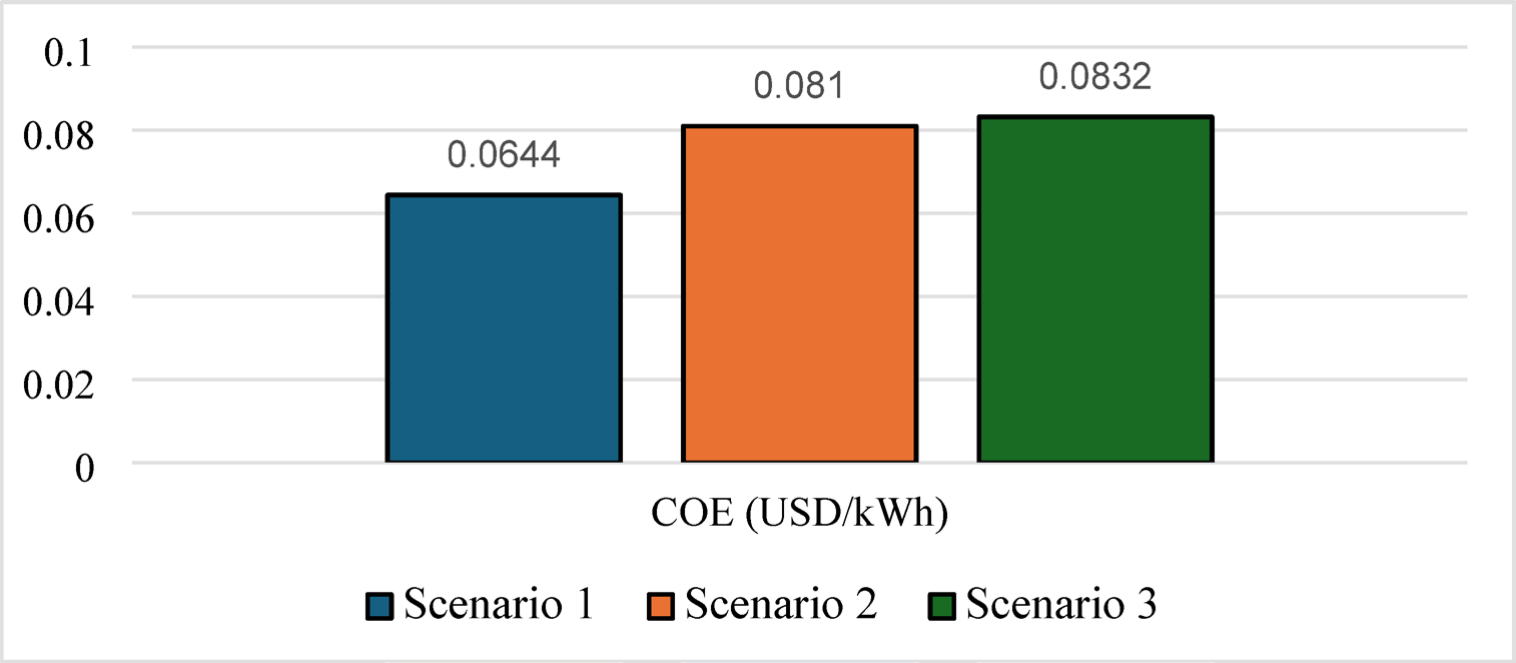
LCOE comparison for the three scenarios.

**Fig. 21:**
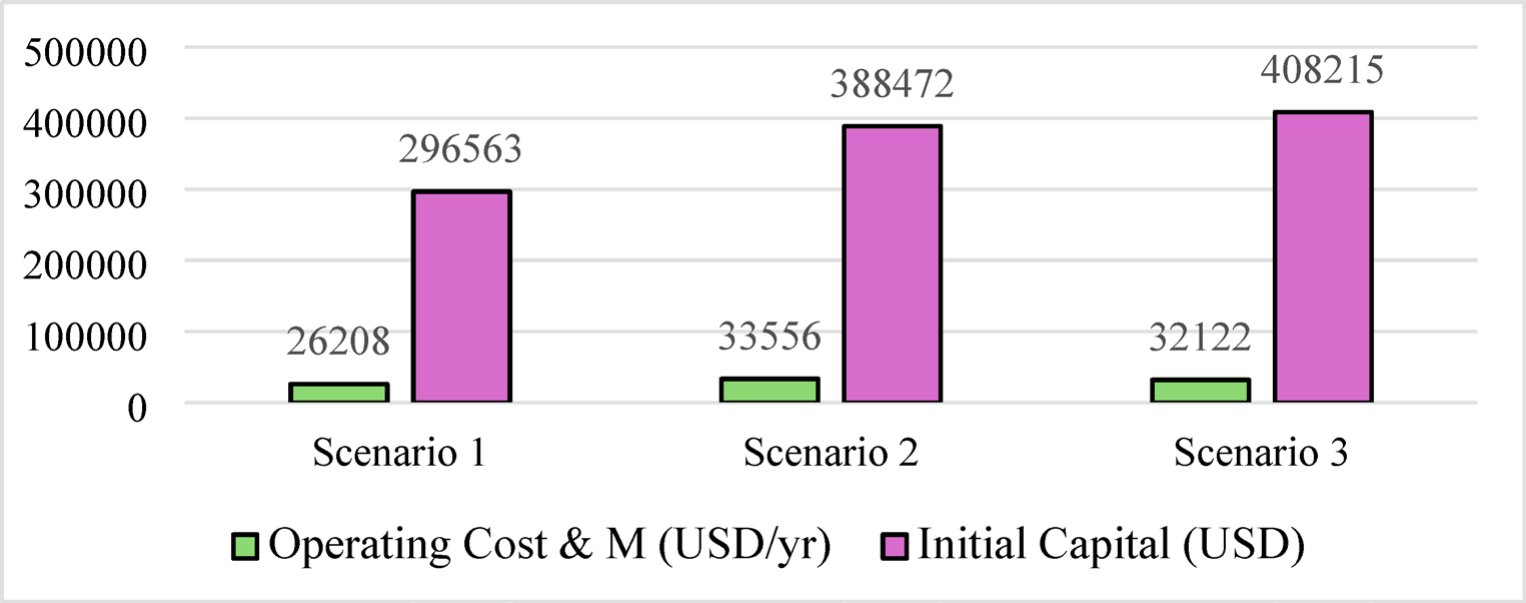
Annual O&M costs and initial capital cost comparison for the three scenarios.

Nevertheless, this superior technical performance comes at a significant cost premium. As shown in Figs. 19 and 20, Scenario 3 has an NPC of 823,477 USD and an LCOE of 0.0832 USD/kWh. Figure 21 further indicates that annual operating and maintenance costs rise to 32,122 USD/yr, reflecting the complexity of managing dual storage, a 100 kW electrolyzer, and a 75 kW fuel cell. While Scenario 1 remains the economic optimum, Scenario 3 delivers superior strategic value in energy security, resilience against grid instability and seasonal stress, and environmental performance. For institutions prioritizing long-term sustainability and carbon neutrality in harsh climates like Sohag, this integrated hybrid configuration represents the only viable pathway to near-100% renewable reliability.

### Performance indicators discussion

The optimization results presented in Tables 4 and 5 demonstrate a clear trade-off between economic performance, renewable energy penetration, and system reliability. Scenario 1 achieved the lowest NPC and LCOE because it relies primarily on the electrical grid and uses only a small battery bank with 100 kWh capacity. The HOMER optimizer selected only one battery unit because the grid is available as a low-cost backup source, making it economically unnecessary to invest in large-scale storage. However, this limited storage capacity resulted in only 1.05 h of battery autonomy, which explains the higher annual grid purchased and the presence of unmet load during periods of low sold generation.

**Table 4:** Optimized system sizing for the three scenarios.

Component	Scenario 1	Scenario 2	Scenario 3
PV (kW)	306	306	308
Battery bank (kWh)	100	No	100
Electrolyzer (kW)	No	100	100
H_2_ tank (kg)	No	50	50
Fuel cell (kW)	No	0	75
Converter (kW)	306	288	309
Dispatch strategy	LF	CC	LF

**Table 5:** Summary of key technical and economic results.

Metric	Scenario 1	Scenario 2	Scenario 3
Renewable fraction (%)	74.7	72.3	75.7
PV production (kWh/yr)	590,959	586,912	628,445
H_2_ production (kg/yr)	0	422	1354
Grid purchased (kWh/yr)	192,890	217,239	193,877
Grid sold (kWh/yr)	92,841	115,029	95,931
Battery autonomy (hr)	1.05	0	1.05
Operation and maintenance (USD/yr)	26,208	33,556	32,122
Initial capital (USD)	296,563	388,472	408,215
NPC (USD)	635,363	822,262	823,477
LCOE (USD/kWh)	0.0644	0.081	0.0832

Scenario 2: The optimizer replaced the battery with a hydrogen subsystem consisting of a 100 kW electrolyzer and a 50 kg hydrogen tank. Although hydrogen storage provides long-duration energy buffering, the round-trip efficiency of the hydrogen cycle is substantially lower than that of batteries. As a result, the system required a higher annual grid purchased (217,239 kWh/yr) despite having similar PV capacity to Scenario 1. Moreover, the optimizer did not select a fuel cell capacity because, under the current electricity tariffs, purchasing electricity from the grid was less expensive than reconverting stored hydrogen into electricity. This explains why Scenario 2 produced only 422 kg/yr of hydrogen and exhibited a lower renewable fraction than Scenario 1.

The selection of dispatch strategies by the HOMER optimizer reflects as shown in Table 4 the structural differences between the investigated scenarios. Scenario 2 relies solely on the hydrogen subsystem for energy storage and does not include a battery for short-term balancing. Under these conditions, the CC strategy becomes more suitable, as it allows the electrolyzer to operate at or near its rated capacity during periods of excess generation. This enables more effective hydrogen production and reduces frequent start-stop operation, which would otherwise decrease system efficiency. In contrast, Scenarios 1 and 3 include battery storage, which provides sufficient flexibility for short-term load matching, making the LF strategy more efficient and economically favorable.

Scenario 3 demonstrates the strongest overall technical performance because it combines the complementary strengths of batteries and hydrogen storage. The optimizer increased the PV size to 308 kW to provide sufficient surplus electricity for both direct load supply and hydrogen production. The reduction in grid purchased from 217,239 kWh/yr in Scenario 2 to 193,877 kWh/yr in Scenario 3 can therefore be attributed to the synergistic interaction between short‑term battery storage and long‑term hydrogen storage. Batteries absorb rapid load fluctuations and improve dispatch flexibility, while hydrogen serves as a seasonal or long‑duration reserve during prolonged low‑generation periods. This hybrid storage strategy also explains the substantial increase in hydrogen production to 1354 kg/yr and the near‑elimination of unmet load (effectively zero). Despite its superior reliability and renewable penetration, Scenario 3 exhibited the highest NPC (823,477 USD) and LCOE (0.0832 USD/kWh) because hydrogen infrastructure remains capital‑intensive. The need for an electrolyzer, fuel cell, storage tank, battery bank, and associated balance‑of‑plant equipment significantly increased the initial investment.

Therefore, the observed trend across the three scenarios reflects an upward shift from economic optimization toward resilience optimization. Scenario 1 is economically attractive but less reliable; Scenario 2 highlights the limitations of small-scale hydrogen-only systems, while Scenario 3 was designed primarily to maximize reliability and energy independence rather than minimize cost. So, the high LCOE in Scenario 3 should not be interpreted solely as an economic disadvantage. Rather, it reflects the cost of resilience, where the system sacrifices short-term economic performance in exchange for higher renewable energy penetration, near-zero unmet load, reduced grid dependence, and improved long-term energy security.

Similar trends have been reported in the literature, where systems integrating both battery and hydrogen storage generally achieve higher renewable penetration and reliability, but at the expense of a higher LCOE and NPC. For example, a recent campus-based hybrid hydrogen study in Turkey reported that grid-connected PV/wind/hydrogen systems achieved a very low energy cost because the grid was heavily utilized as a balancing source. In contrast, systems with larger hydrogen storage capacities required substantially higher investments in electrolyzers, storage tanks, and conversion equipment. This finding is consistent with Scenario 3 of the present study, where the addition of an electrolyzer, fuel cell, hydrogen tank, and battery bank significantly increased both NPC and LCOE^44^.

Likewise, several published studies concluded that battery-based systems are generally more economical than hydrogen-only systems because batteries have higher round-trip efficiency and lower operational losses. A study on PV/fuel-cell hybrid systems found that configurations including hydrogen storage often have higher lifecycle costs than battery-dominated systems, despite providing better long-term storage capability and improved energy autonomy^45,46^. The present findings also agree with Egyptian studies on hybrid renewable systems, which showed that the incorporation of hydrogen storage can improve renewable utilization and reduce dependence on the grid, but only when the system is sufficiently large to justify the high capital cost of hydrogen infrastructure. Small hydrogen subsystems often underperform economically because the hydrogen production and reconversion cycle introduces significant energy losses^47,48^.

Moreover, previous comparative studies demonstrated that systems combining batteries with hydrogen storage usually outperform systems relying on only one storage technology. Batteries are more effective for short-term fluctuations and daily load balancing, while hydrogen is better suited for long-duration and seasonal storage. Therefore, hybrid battery-hydrogen configurations can substantially reduce unmet load and increase renewable fraction, which explains why Scenario 3 achieved the highest technical performance in the current study^49,50^. Accordingly, the results of the present work are consistent with the broader literature: lower-cost systems tend to rely more on grid electricity and batteries, while highly resilient systems with greater renewable penetration require hydrogen infrastructure and therefore exhibit higher LCOE values.

To complement the renewable fraction analysis, annual CO_2_ emissions were extracted from the HOMER optimization outputs for each scenario, as presented in Table 6. Using the Egypt grid emission factor of 0.45 kg CO_2_/kWh based on the EgyptERA 2024/2025 fiscal year data.

**Table 6:** Annual emissions and CO_2_ reduction for each scenario.

Scenario	Annual CO_2_ emissions (kg/yr)	CO_2_ avoided vs. Grid-only baseline (kg/yr)	Reduction (%)
Grid-only baseline	2,722,000	–	–
Scenario 1: PV/BESS/UG	121,907	2,600,093	95.5%
Scenario 2: PV/FC/H_2_/UG	135,139	2,586,861	95%
Scenario 3: PV/FC/BESS/H_2_/UG	122,530	2,599,470	95.5%

The grid-only baseline supplying the full annual demand of 6048 MWh would emit approximately 2722 tonnes of CO_2_ per year. While the differences between scenarios are relatively small in percentage terms due to the dominant role of PV generation across all configurations, the strategic advantage of Scenario 3 lies in its superior reliability rather than marginal emission reduction. Over the 25-year project lifetime, Scenario 3 would cumulatively avoid approximately 64,987 tonnes of CO_2_, underscoring its strategic value for long‑term decarbonization of public infrastructure.

## Validation and modeling

To verify the effectiveness of the integration of the utility grid with the photovoltaic system, battery storage, electrolyzer, hydrogen tank, fuel cell, and inverter, it is imperative to validate the system using a globally recognized testbed. The IEEE 33-bus radial distribution network was selected for this purpose, as it serves as a standard benchmark in the power systems community for assessing voltage stability and renewable energy penetration. Testing on this complex architecture ensures that the proposed solution is not only theoretically sound but also practically scalable to real-world distribution grids. The HOMER analysis was first used to determine the optimal component sizes, dispatch strategy, renewable fraction, and techno-economic performance of each hybrid microgrid configuration. The resulting capacities were then used as input parameters in the IEEE 33-bus distribution network model. Therefore, the IEEE 33-bus model was not treated as a separate analysis, but rather as a validation framework used to assess how the HOMER-based optimal configuration would perform when integrated into a realistic distribution network environment.

**Fig. 22:**
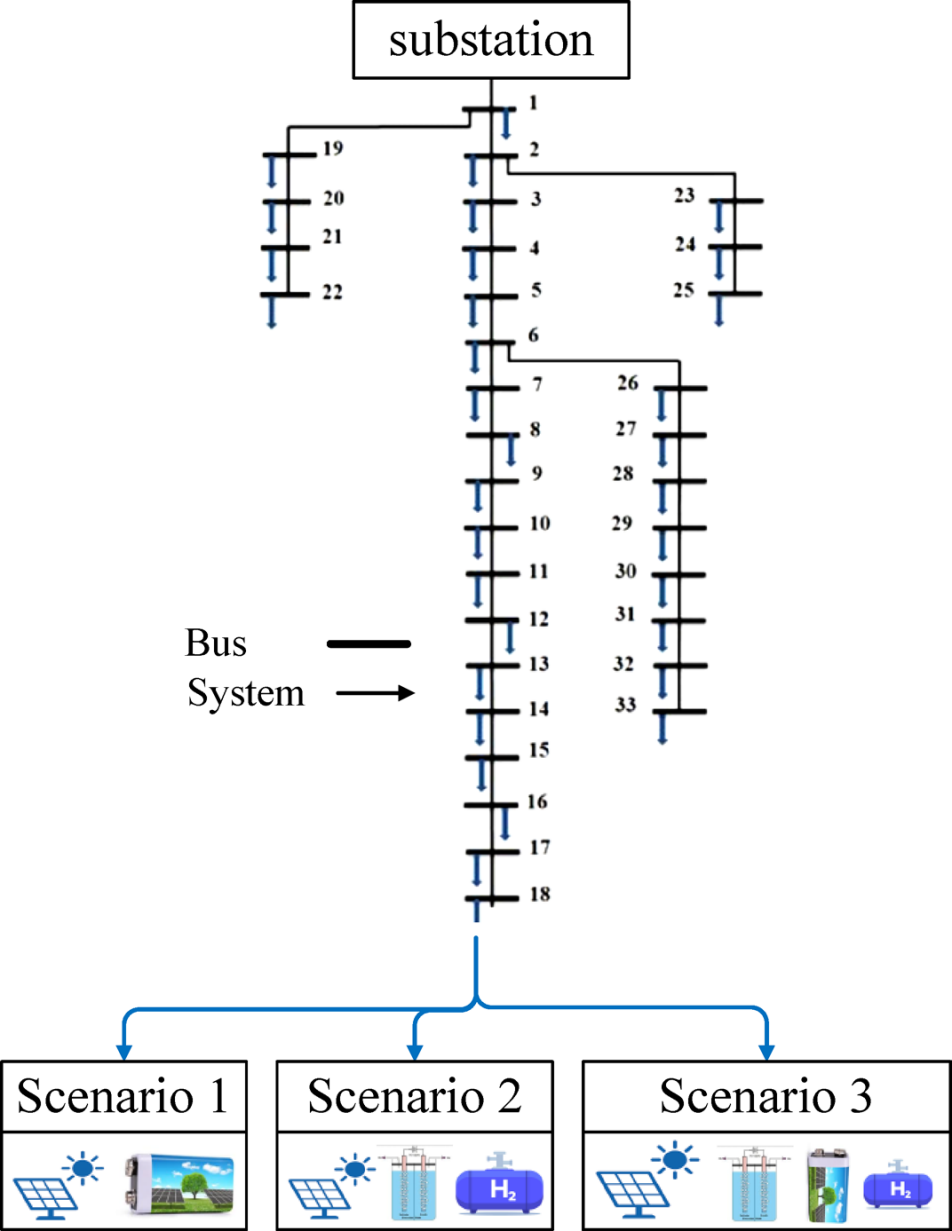
Single-line diagram of the IEEE 33-bus radial distribution system integrated with the three proposed hybrid scenarios at bus 18.

The steady-state analysis and power flow calculations were performed using the forward–backward sweep (FBS) algorithm in MATLAB using the IEEE 33-bus radial distribution system model, as shown in Fig. 22. The FBS was specifically chosen for its superior convergence rate and computational efficiency in radial topologies. By utilizing this algorithm, we can accurately model the recursive relationship between branch currents and nodal voltages, providing a precise evaluation of how strategic power injection at critical nodes can mitigate the inherent voltage drops and support the grid overall reliability.

The impact of the proposed scenarios on the grid voltage stability is illustrated in Fig. 22. To ensure a rigorous evaluation, the analysis followed a structured methodology, starting from power calculation to the final performance assessment.

### Calculation methodology of injected power

The optimized hybrid system output was converted into an equivalent net power injection. ${P}_{inj}$ profile based on the ratio between the studied facility load and the total load of the IEEE 33-bus system. The scaling factor was defined as:

5 $$SF=\frac{{P}_{Load,case}}{{P}_{Load,IEEE}}$$

where:

- ${P}_{Load,case}$: Represents the peak load of the faculty.
- ${P}_{Load,IEEE}$: Corresponds to the total load of the IEEE 33-bus system.

The determination of the injected power at bus 18 was based on the net deficit required to stabilize the nodal voltage. By utilizing the FBS algorithm, the precise amount of power needed to bridge the gap between the actual voltage and the desired 1.0 pu target was identified, ensuring that each scenario provides the exact support required to eliminate the voltage drop detected in the baseline state. In each scenario, ${P}_{inj}$ was calculated as the sum of renewable generation and storage compensation (${P}_{inj,HOMER}={P}_{PV}+{P}_{storage}$). The net injected power from the hybrid system was then scaled accordingly:

6 $${P}_{inj,scaled}={P}_{inj,HOMER}*SF$$

This approach ensures that the relative contribution of the hybrid system is realistically represented within the IEEE test network without overloading or distorting the system characteristics.

### Impact of the three scenarios on the grid

**Fig. 23:**
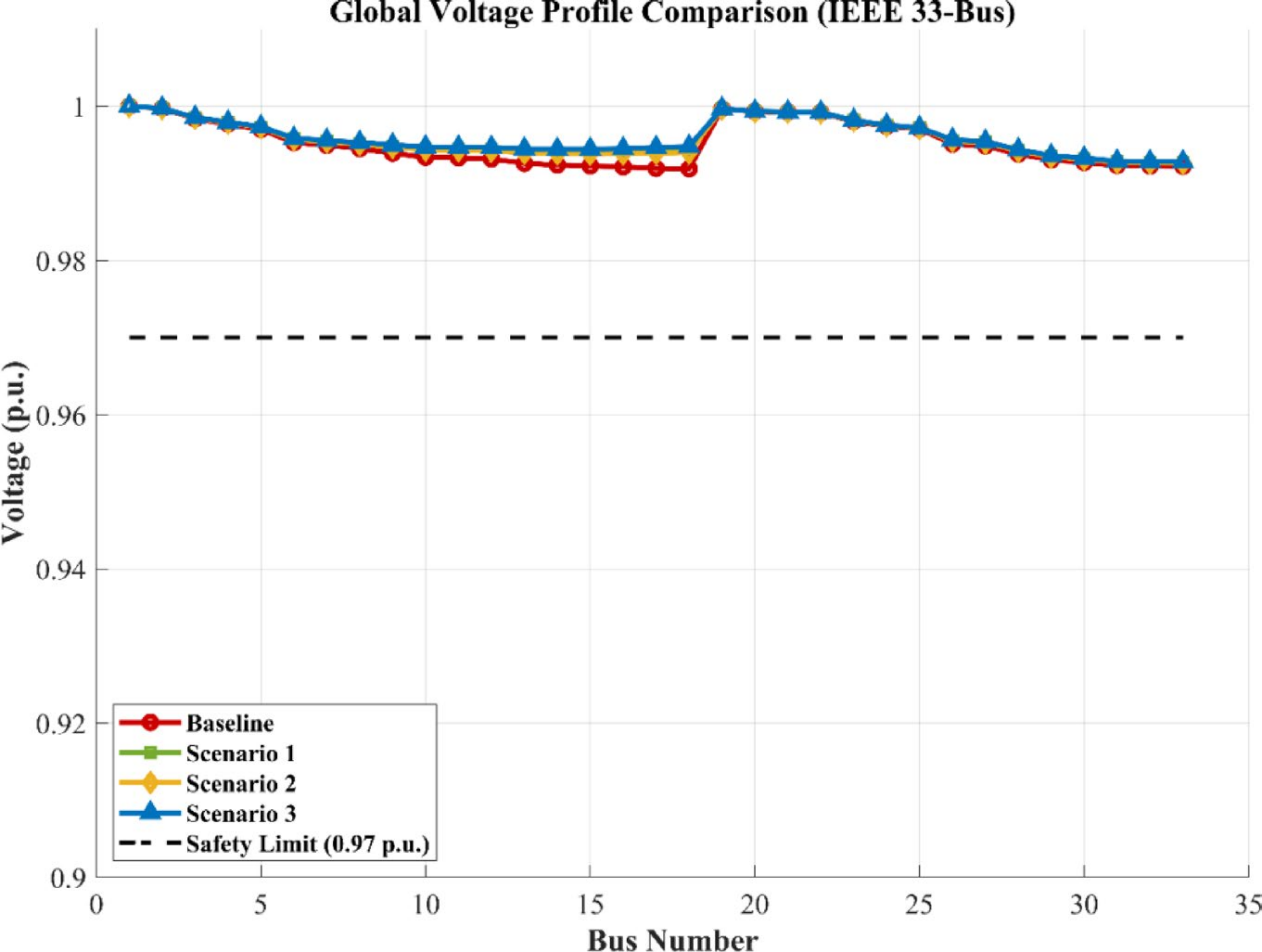
Voltage profile comparison across baseline and three scenarios for the IEEE 33-bus system.

**Fig. 24:**
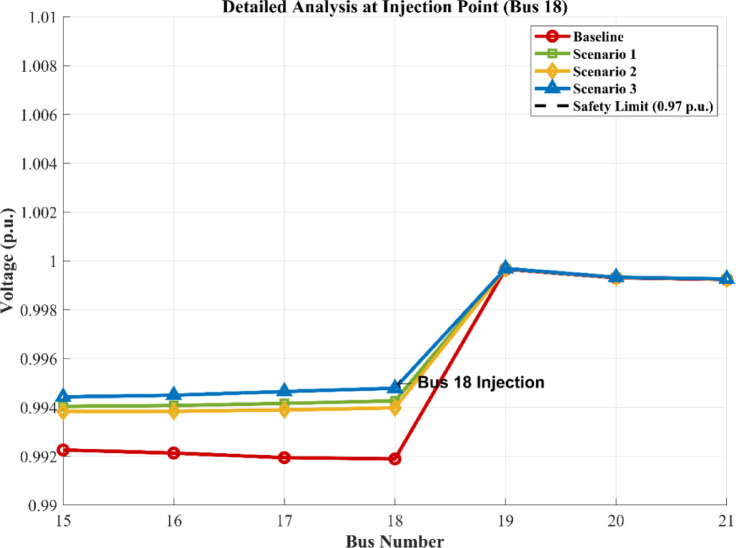
Voltage magnitude at each bus for baseline and three scenarios.

**Fig. 25:**
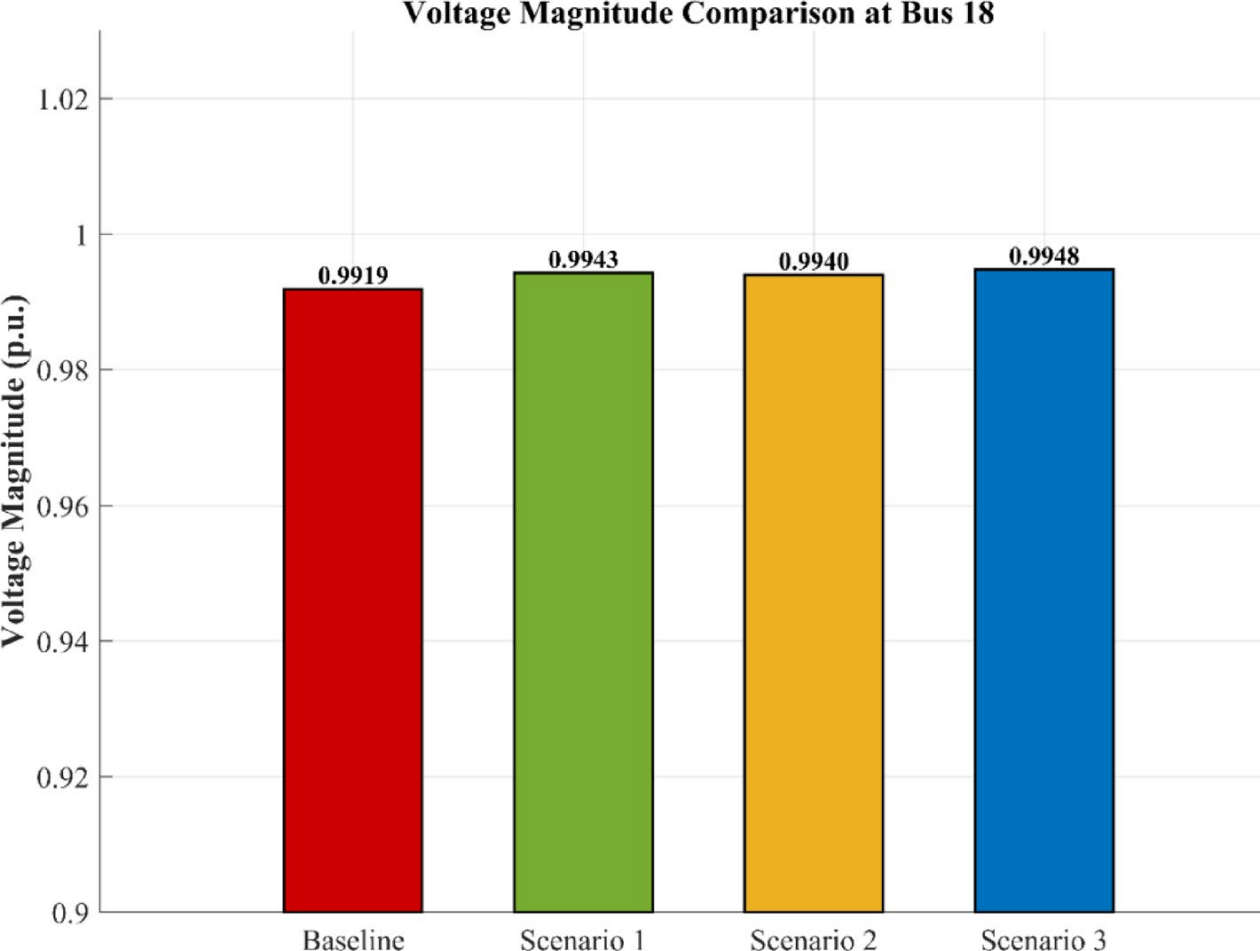
Comparative analysis of voltage magnitude at bus 18 across baseline and hybrid integration scenarios.

As demonstrated in Figs. 23, 24 and 25, the injection of the calculated power resulted in varying degrees of improvement across the network. All scenarios succeeded in shifting the voltage profile upward from the baseline. The scenarios involving single storage integration, Scenario 1 (PV/BESS) or Scenario 2 (PV/H_2_), showed a marked improvement in the voltage levels of the surrounding buses (13–23), effectively mitigating the stress on the radial feeder and enhancing the overall power quality of the IEEE 33-bus system.

### The optimal scenario and the associated cost penalty

Among the tested configurations, Scenario 3 (PV/BESS/H_2_) represented by the blue curve in Fig. 24 emerged as the superior technical choice. This scenario achieved the most stable and ideal voltage profile, providing a robust buffer against fluctuations. However, it is imperative to acknowledge the cost penalty associated with this optimal performance. The transition to this integrated hybrid model involves significantly higher initial capital expenditure due to the complexity of combining hydrogen fuel cells with battery storage. Therefore, while Scenario 3 offers the maximum technical advantages for the grid, it requires a higher financial investment compared to simpler configurations.

## Conclusion and future work

This study conducted a comprehensive techno-economic assessment and grid-level validation of hybrid PV/hydrogen/battery storage systems to supply an academic building under the hyper-arid climatic conditions of Sohag, Upper Egypt. The comparative analysis of three grid-connected scenarios revealed a clear and quantifiable trade-off between economic optimality and energy resilience. While Scenario 1 (PV/BESS/UG) achieved the lowest LCOE at 0.0644 USD/kWh and the lowest NPC of 635,363 USD, its limited battery autonomy of only 1.05 h resulted in persistent grid dependence and unmet loads during peaks. Conversely, Scenario 2 (PV/FC/H_2_/UG), which relied solely on hydrogen storage, underperformed economically, with the optimizer avoiding fuel cell dispatch altogether due to the high cost of reconversion, leading to a 12.6% increase in grid purchased and a reduced renewable fraction of 72.3%. The integrated hybrid system Scenario 3: (PV/FC/BESS/H_2_/UG) emerged as the most technically superior configuration, achieving the highest renewable fraction 75.7%, near-zero unmet load LPSP effectively zero (i.e., below the simulation tolerance), LOLP = 0.00%, and substantial green hydrogen production 1354 kg/year. These reliability indices quantitatively confirm that the hybrid system achieves 100% supply reliability under the modeled conditions. However, this resilience came at a significant cost penalty, with the highest NPC (823,477 USD) and LCOE (0.0832 USD/kWh). Validation using the IEEE 33-bus radial distribution network and the forward–backward sweep algorithm confirmed that strategic power injection from this hybrid configuration, particularly at Bus 18, effectively stabilized voltage profiles near 1.0 p.u., demonstrating tangible grid-support capabilities. This research concludes that while battery-only systems remain economically preferable under current tariffs, the synergy between short-term battery storage and long-term hydrogen storage is indispensable for achieving energy independence, grid stability, and alignment with net-zero emissions targets in hot, off-grid-prone environments such as Upper Egypt. The higher cost of resilience should therefore be interpreted not as an economic drawback, but as a strategic investment toward sustainable, zero-emission public infrastructure.

Future research should extend the present analysis in three key directions: First, while the sensitivity analysis examined tariff and cost variations, a probabilistic Monte Carlo approach is needed to capture the combined uncertainty of exchange rates, carbon pricing, and solar resource variability under Egypt Vision 2030. Second, rapid cost reductions in PEM electrolysis and fuel cells projected to fall from ~ 800–1200 USD/kW to 400–600 USD/kW by 2030 could lower the NPC of Scenario 3 by 12–15%. Future studies should incorporate learning curves to identify the break‑even year when green hydrogen becomes cost‑competitive with grid purchases. Third, long‑term operational validation through a 3–5 year pilot installation is essential to quantify actual degradation of batteries and fuel cells, maintenance requirements, and dust‑related PV losses under Sohag hyper‑arid conditions. Additionally, dynamic grid stability studies using MATLAB/Simulink and predictive dispatch strategies based on solar forecasting would further validate the system resilience. These efforts will transform the present optimization into a deployable model for net‑zero public infrastructure in hot, grid‑stressed regions.

## Data Availability

Data are available on request from the authors.

## Abbreviations

AC: Alternating current
AEL: Alkaline electrolysis
BESS: Battery energy storage system
BOP: Balance of plant
CAPEX: Capital expenditure
CC: Cycle charging
CPVT: Concentrated photovoltaic thermal
DC: Direct current
DOD: Depth of discharge
EGP: Egyptian pound
EgyptERA: Egypt electric utility and consumer protection regulatory agency
FBS: Forward-backward sweep
FC: Fuel cell
GHI: Global horizontal irradiance
H_2_: Hydrogen storage
HESS: Hybrid energy storage system
HHV: Higher heating value
HOMER: Hybrid optimization model for multiple energy resources
HVAC: Heating, ventilation, and air conditioning
IRENA: International renewable energy agency
LCOE: Levelized cost of energy
LF: Load following
LHV: Lower heating value
LLP: Loss of load probability
NASA: National aeronautics and space administration
NOCT: Nominal operating cell temperature
NPC: Net present cost
O&M: Operation and maintenance
OPEX: Operational expenditure
ORC: Organic Rankine cycle
PEMEL: Proton exchange membrane electrolysis
Pinj: Injected power
PV: Photovoltaic
SOC: State of charge
SOEL: Solid oxide electrolysis
UG: Utility grid
USD: United States dollar
